# Tumor therapy by targeting extracellular hydroxyapatite using novel drugs: A paradigm shift

**DOI:** 10.1002/cam4.6812

**Published:** 2024-01-18

**Authors:** Mohammed N. Tantawy, J. Oliver McIntyre, Fiona Yull, M. Wade Calcutt, Dmitry S. Koktysh, Andrew J. Wilson, Zhongliang Zu, Jeff Nyman, Julie Rhoades, Todd E. Peterson, Daniel Colvin, Lisa J. McCawley, Jerri. M. Rook, Barbara Fingleton, Marta Ann Crispens, Ronald D. Alvarez, John C. Gore

**Affiliations:** ^1^ Vanderbilt University Institute of Imaging Science Vanderbilt University Medical Center Nashville Tennessee USA; ^2^ Departments of Radiology and Radiological Sciences Vanderbilt Univerity Medical Center Nashville Tennessee USA; ^3^ Department of Pharmacology Vanderbilt University Nashville Tennessee USA; ^4^ Department of Obstetrics and Gynecology Vanderbilt Univerity Medical Center Nashville Tennessee USA; ^5^ Department of Biochemistry Vanderbilt University Nashville Tennessee USA; ^6^ Mass Spectrometry Research Center of Chemistry Vanderbilt University Nashville Tennessee USA; ^7^ Department of Chemistry Vanderbilt University Nashville Tennessee USA; ^8^ Vanderbilt Institute of Nanoscale Science and Engineering Vanderbilt University Nashville Tennessee USA; ^9^ Department of Biomedical Engineering Vanderbilt University Nashville Tennessee USA; ^10^ Orthopaedic Surgery Vanderbilt Univerity Medical Center Nashville Tennessee USA; ^11^ Department of Veterans Affairs, Tennessee Valley Healthcare System Nashville Tennessee USA; ^12^ Division of Gynecologic Oncology Vanderbilt Univerity Medical Center Nashville Tennessee USA

**Keywords:** breast cancer, hydroxyapatite, PET, SPECT, tumor extracellular pH, tumor metabolism, tumor microenvironment

## Abstract

**Background:**

It has been shown that tumor microenvironment (TME) hydroxyapatite (HAP) is typically associated with many malignancies and plays a role in tumor progression and growth. Additionally, acidosis in the TME has been reported to play a key role in selecting for a more aggressive tumor phenotype, drug resistance and desensitization to immunotherapy for many types of cancers. TME‐HAP is an attractive target for tumor detection and treatment development since HAP is generally absent from normal soft tissue. We provide strong evidence that dissolution of hydroxyapatite (HAP) within the tumor microenvironment (TME‐HAP) using a novel therapeutic can be used to kill cancer cells both *in vitro* and *in vivo* with minimal adverse effects.

**Methods:**

We developed an injectable cation exchange nano particulate sulfonated polystyrene solution (NSPS) that we engineered to dissolve TME‐HAP, inducing localized acute alkalosis and inhibition of tumor growth and glucose metabolism. This was evaluated in cell culture using 4T1, MDA‐MB‐231 triple negative breast cancer cells, MCF10 normal breast cells, and H292 lung cancer cells, and *in vivo* using orthotopic mouse models of cancer that contained detectable microenvironment HAP including breast (MMTV‐Neu, 4T1, and MDA‐MB‐231), prostate (PC3) and colon (HCA7) cancer using ^18^F‐NaF for HAP and ^18^F‐FDG for glucose metabolism with PET imaging. On the other hand, H292 lung tumor cells that lacked detectable microenvironment HAP and MCF10a normal breast cells that do not produce HAP served as negative controls. Tumor microenvironment pH levels following injection of NSPS were evaluated via Chemical Exchange Saturation (CEST) MRI and via *ex vivo* methods.

**Results:**

Within 24 h of adding the small concentration of 1X of NSPS (~7 μM), we observed significant tumor cell death (~ 10%, p < 0.05) in 4T1 and MDA‐MB‐231 cell cultures that contain HAP but ⟨2% in H292 and MCF10a cells that lack detectable HAP and in controls. Using CEST MRI, we found extracellular pH (pHe) in the 4T1 breast tumors, located in the mammary fat pad, to increase by nearly 10% from baseline before gradually receding back to baseline during the first hour post NSPS administration. in the tumors that contained TME‐HAP in mouse models, MMTV‐Neu, 4T1, and MDA‐MB‐231, PC3, and HCA7, there was a significant reduction (*p*<0.05) in ^18^F‐Na Fuptake post NSPS treatment as expected; ^18^F^‐^ uptake in the tumor = 3.8 ± 0.5 %ID/g (percent of the injected dose per gram) at baseline compared to 1.8 ±0.5 %ID/g following one‐time treatment with 100 mg/kg NSPS. Of similar importance, is that ^18^F‐FDG uptake in the tumors was reduced by more than 75% compared to baseline within 24 h of treatment with one‐time NSPS which persisted for at least one week. Additionally, tumor growth was significantly slower (*p* < 0.05) in the mice treated with one‐time NSPS. Toxicity showed no evidence of any adverse effects, a finding attributed to the absence of HAP in normal soft tissue and to our therapeutic NSPS having limited penetration to access HAP within skeletal bone.

**Conclusion:**

Dissolution of TME‐HAP using our novel NSPS has the potential to provide a new treatment paradigm to enhance the management of cancer patients with poor prognosis.

## Graphical abstract

We have developed a cation ion nanoparticulate sulfonated polystyrene solution (NSPS) to target and chelate tumor microenvironment hydroxyapatite (TME‐HAP) releasing phosphate and hydroxyl ions in the TME and subsequently inducing acute localized alkalosis. Chelation of TME‐HAP with NSPS resulted in the inhibition of tumor cell metabolism and proliferation leading to tumor cell apoptosis in mouse models. There are no other known injectable compounds that can break up TME‐HAP in vivo making NSPS a one‐of‐a‐kind and first in a class of novel cancer therapeutics.

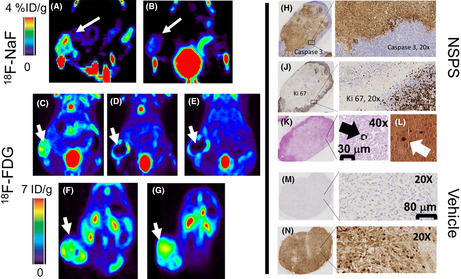

## 1 INTRODUCTION

Hydroxyapatite (HAP), Ca_10_(PO_4_)_6_OH_2_, once thought to be solely a ubiquitous component of bone and teeth, has also been shown to be produced by malignancies in cell cultures and in vivo.^1^, ^2^, ^3^, ^4^, ^5^, ^6^, ^7^, ^8^, ^9^, ^10^, ^11^, ^12^, ^13^, ^14^, ^15^ Formation of such tumor‐associated HAP is postulated to involve alkaline phosphatase (ALP) mediated release of inorganic phosphate (Pi) that then combines with calcium to produce HAP crystals. These crystals are then deposited in the extracellular matrix and subsequently influence the tumor microenvironment (TME).^3^ TME‐HAP has been shown to enhance tumor proliferation, progression, and migration by promoting mitogenesis and matrix metalloproteinase (MMPs) expression.^1^, ^2^, ^3^, ^4^, ^5^, ^6^, ^7^, ^8^, ^9^, ^16^ MMPs promote tumor growth by degrading matrix barriers and by enhancing angiogenesis.^3^, ^17^ We have shown in previous work that HAP‐binding radiotracers such as FDA‐approved ^18^F‐labeled sodium fluoride (^18^F‐NaF) and ^99m^Tc‐labeled methyl diphosphate (^99m^Tc‐MDP) could be used with positron emission tomography (PET) and single photon emission computed tomography (SPECT), respectively, to detect breast tumors, gastric tumors, and peritoneal ovarian tumors^11^, ^12^, ^15^, ^18^, ^19^ that contain HAP in the tumor microenvironment. Detection of tumor‐associated HAP exhibited high specificity and high signal‐to‐background ratio (SBR) as HAP is absent in normal soft tissues.^11^, ^12^, ^15^, ^18^, ^19^ This strategy has subsequently been used by others for detecting primary breast tumors^13^ and hepatic.

Triple negative breast cancer (TNBC), lacking estrogen, progesterone and HER2 receptors, is usually more aggressive, harder to treat, associated with chemoresistance, high recurrence rates, distant metastases, and poor overall survival (OS) compared to cancers that are hormone receptor and/or HER2 positive.^20^, ^21^, ^22^, ^23^, ^24^, ^25^, ^26^ Acidosis in the tumor microenvironment may play a role in alteration of drug structure and/or uptake, contributing to resistance to cytotoxic chemotherapy.^27^, ^28^ Additionally, acidosis may result in elevated expression of programmed death ligand 1 (PD‐L1),^29^ and diminished activity of cytotoxic T lymphocytes (CTLs).^30^ There is an urgent clinical need for new treatment paradigms that could improve the outcome for cancer patients with poor prognosis.

We have now successfully engineered *a* novel injectable cation exchange nanoparticulate sulfonated polystyrene solution (NSPS) designed to breakup TME‐HAP and increase tumor extracellular pH (pHe) by chelating calcium, similar to cation exchange resins:

$$\text{2NSPS}+{\mathrm{Ca}}^{++}⇌{\text{NSPS}}_{2}\mathrm{Ca}+{\text{2Na}}^{+}\;\;\left(\text{Equation}\;1\right)$$

and in return releasing (PO_4_)^−^ and OH^−^ anions. We provide compelling evidence of the efficacy of NSPS in the treatment of tumors with TME‐HAP in murine models. While we had communicated preliminary results to one of our funding agencies, Department of Defense Ovarian Cancer Research Program (DoD OCRP), to our knowledge, this the first publication that reports the exploitation of TME‐HAP as a therapeutic target. TME‐HAP is absent from normal soft tissue but produced directly by tumor cells^1^, ^2^, ^3^, ^4^, ^5^, ^7^, ^8^, ^9^, ^10^, ^16^ and is distinct from exogenously administered manufactured ultra fine nano HAP (nHAP) injected directly into tumors or used as a vehicle for drug delivery.^31^, ^32^, ^33^, ^34^, ^35^, ^36^, ^37^, ^38^, ^39^

## 2 METHODS

### 2.1 Preparation of NSPS

Six hundred milliliters (600 mL) of deionized water were added to 600 g of Amberlite IR120 Na^+^ (Sigma Aldrich) beads +6 mL of 0.9% clinical grade saline (Vanderbilt University Medical Center, VUMC, Pharmacy); that is, 1:1:0.01 mixture. The mix was stirred at 650 rotations per minute for at least 5 days or until the mixture had a milk‐like white appearance. Then, the mixture was filtered through a 0.2 micrometer membrane (VWR, Suwanee GA USA) and allowed to air dry in a bio‐hood. The dried resolute which weighed 2.4 ± 0.3 mg per 5 mL filtered mixture, was then mixed with 0.02 mL biograde ethanol (96% bio‐EtOH) (Sigma Aldrich) and 0.2 mL saline. This final formulation is what we refer to as a nanoparticulate sulfonated polystyrene solution (NSPS) which was used for characterization and preclinical tests.

### 2.2 Properties of NSPS

Triplicate independent samples of NSPS were analyzed by:

- Fourier Transform Infrared Spectra (FT‐IR) and nuclear magnetic resonance (NMR) spectra were carried out by EAG Laboratories (Maryland Heights, MS) on triplicate preparations of NSPS and the solute following evaporation of the water; NMR spectral analysis was also confirmed by the Vanderbilt Small Molecule NMR Facility Core (Vanderbilt University School of Medicine, Nashville, TN).
- Mass spectrometry: high resolution mass spectrometry was performed in the Vanderbilt Mass Spectrometry Core Facility. Samples were analyzed by direct liquid infusion using an Orbitrap mass spectrometer (Thermo‐Finnigan) equipped with an Ion‐Max source housing and a standard electrospray (ESI) ionization probe in positive and negative ion modes at a resolving power of 60,000 (at *m/z* 400).
- Particle size and distribution parameters were measured by dynamic light scattering (DLS) using a DLS Malvern Nano ZS instrument (Malvern Instruments) in the Vanderbilt Institute of Nanoscale Science and Engineering (ViNSE). This step was repeated in triplicates 3 times on 3 different preparations of NSPS, that is, *N*_total_ = 9.
- Ultraviolet absorption (UV) spectroscopy: UV absorbance spectra were obtained using a UV‐2501PC dual‐beam spectrophotometer (Shimadzu) with 10 mm pathlength quartz cuvettes.

Based on the results, we identified a novel structure that we postulate to be the cytotoxic active ingredient in NSPS. Therefore, we synthesized a novel sulfonated benzene monomer (VU0945652) with mass of 199 Daltons at the Vanderbilt Chemical Synthesis Core from 3‐bromobenzenesulfonic acid and potassium vinyl trifluoroborate using palladium cross‐coupling reaction.

### 2.3 NSPS impact on calcium phosphates phantoms

To test our theory that NSPS increases pH by dissolution of TME‐HAP, we filled phantoms with 10 mL of deionized water +1 mg of calcium phosphates salt (Thermo Fisher) + either 0.2 mL of 1× NSPS (i.e., concentration of NSPS = 4.27 nmol/mL for 55 kDa molecular weight) or vehicle (0.2 mL saline). The pH of the phantoms was measured using a standard pH meter (Vernier Go Direct) at baseline and at 24 h and 7 days post NSPS/vehicle addition.

### 2.4 Assessing the In vitro efficacy of NSPS

NSPS efficacy was tested using two tumorigenic breast cell lines that produce extracellular matrix HAP (ECM‐HAP); mouse 4 T1^3^ and MDA‐MB‐231.^11^, ^12^ In addition, MCF10a normal mammary epithelial cell line that does not produce detectable ECM‐HAP^3^ were used. We found that H292 lung tumors in xenograft models and in cell culture lacked detectable TME‐HAP or ECM‐HAP. Therefore, H292 tumors serve as a negative control, in vivo, while the MCF10a cell line serves as a suitable negative control in vitro. Cells were cultured in plates using 10% FBS‐supplemented DMEM glucose rich medium or RPMI‐1640 (for H292) with 1% penicillin/streptomycin. Additionally, the 4 T1 culture contained an osteogenic cocktail consistent of 50 μgmL^−1^ ascorbic acid and 10 μM β‐glycerophosphate (βG) as recommended by Cox and others for at least 11 days.^3^, ^10^ Cells grown in osteogenic cocktail exhibited changes in morphology consistent with those reported by Morgan and colleagues.^3^, ^10^ When each of the cell lines reached ~70% confluency, they were split into 24 wells (1 mL per well).

NSPS (X = 2.4 mg in 0.02 mL Bio‐EtOH +0.2 mL saline) was diluted with 6 mL of the culture medium, that is, concentration of NSPS = 7.02 nmol/mL (~7 μM) for 55 kDa molecular weight. For controls, we mixed vehicle (0.2 mL saline +0.02 mL bio‐EtOH) with 6 mL of the culture media. When all cells reached ~70% confluency in the 24 wells, we aspirated the original media and then added 1 mL of the media + NSPS or vehicle. About 18 h later, we measured the number of total cells and dead cells using a hemocytometer and trypan blue. The test was repeated on the 4 T1 cultures at 0.3 X concentration of NSPS.

To verify that active ingredient in NSPS is VU0945652, we repeated the above study except this time, X = 20 mg of VU0945652 were mixed with 0.02 mL bio‐EtOH +0.2 mL saline with 6 mL culture media (16.2 μmol/mL). Then we added 1 mL of VU0945652 + media or vehicle + media to each well in a 24 well plate containing 4 T1 breast cultured in osteogenic media or H292 lung cultured in RPM1400. The test was repeated on the 4 T1 cultures at 0.3 X concentration of VU0945652.

Analysis: We conducted statistical comparisons between treatment and control (vehicle) cell cultures using unpaired two‐tailed *t*‐tests for each treatment type (NSPS, VU0945652), each treatment dose, and each cell line.

### 2.5 In vivo studies with NSPS

#### 2.5.1 Mouse models

All animal studies were approved by the Vanderbilt University Institutional Animal Care and Use Committee (IACUC). We used the following mouse models for studying the mechanisms of action and efficacy of NSPS:

1‐MDA‐MB‐231 human breast cells (1 × 10^6^) were injected in the flank of immunocompromised female athymic nu/nu mice (8 weeks; *n* = 12) similar to previous work.^11^, ^12^, ^40^

2– 4 T1 mouse breast cells (5 × 10^5^) were injected in the mammary fat pad of immunocompetent female Balbc/6 mice (8 weeks; *n* = 10) under the 4th nipple.

3‐MMTV‐Neu breast cells (1 × 10^6^) were surgically placed on the mammary fat pad of immunocompetent syngeneic FVB/n mice (8 weeks; *n* = 7) under the 4th nipple similar to previous work.^41^

4‐H292 human lung cells (5 × 10^6^) were injected in the flank of immunocompromised female athymic nu/nu mice (8 weeks; *n* = 8).

5‐PC3 human prostate cells (5 × 10^6^) were injected in the flank of immunocompromised male athymic nu/nu mice (8 weeks; *n* = 8).

6‐HCA‐7 colon cells (1 × 10^6^) were injected in the flank of immunocompromised male athymic nu/nu mice (8 weeks; *n* = 8).

Studies were commenced when tumor size was at least 200 mm^3^ as measured by calipers. The length of time after engraftment to achieve this size depended on the model and ranged from 2 weeks (e.g., 4 T1) to approximately 10 weeks (e.g., H292).

### 2.6 Tumor extracellular pH (pHe) measurement, in vivo

All animal procedures were approved by our Institution's Animal Care and Usage Committee.

For in vivo assessment of tumor pHe, 4 T1 breast or H292 lung tumor bearing mice (*n* = 4 per group; NSPS vs vehicle saline) described above were imaged via magnetic resonance imaging (MRI) at 7 Tesla using the chemical exchange saturation transfer (CEST) approach, an established method for measuring extracellular pH ^42^, ^43^, ^44^, ^45^, ^46^, ^47^ in vivo. Clinical grade iohexol; 300 mg/mL Iodine (300 mgI/mL Omnipaque, GE Healthcare, purchased from VUMC pharmacy) was injected (0.2 mL) intraperitoneally and another 0.2 mL were injected directly into the tumors similar to Chen et al^43^ The mice were scanned at baseline and immediately following an intravenous injection, via jugular vein catheter, of either a test dose of 25 mg/kg (0.6 mg) NSPS in 0.05 mL saline or vehicle (0.05 mL saline). The doses were administered after a 0.05 mL saline pre‐flush and followed by an additional 0.05 mL saline flush post NSPS or vehicle injection. The mice were then scanned up to 9 more times using the same CEST MRI protocol. Details of the CEST MRI protocol are provided in supplementary data. Each scan, whether baseline or post NSPS/veh injection, lasted 7.25 min. The injection of NSPS/veh took less than 60 s before the post treatment CEST scans started. The raw CEST data were fitted with a Lorentzian curve and compared to phantoms using the same MRI protocol. Details of the Lorentzian fitting approach are outlined in the supplementary data. The phantoms were prepared by mixing 0.2 mL of Omnipaque 300 with 1 mL deionized H_2_O and NaOH or HCL was added in 0.005 mL aliquots to adjust the pH. Seven phantoms with pH ranging between 6.15 and 9.15 were then scanned with the same CEST MRI protocol. Since iohexol has only one peak at ~4 ppm making it difficult to obtain absolute quantification of pH, we used the phantom as a map to determine the direction of change of pH in vivo.

For ex vivo pH analysis, groups of 4 T1 orthotopic tumor bearing mice (*n* = 3 per group) were treated with 25 mg/kg/0.05 mL NSPS, i.v., 0.05 mL vehicle (saline), or no treatment. Within 20–30 min, the tumors were harvested and homogenized. The pH of the homogenates were measured directly using a Verner pH meter. The meter was thoroughly washed in deionized water and wiped clean between each reading.

### 2.7 In vivo efficacy of NSPS

MMTV‐Neu breast tumor mice received an intravenous (i.v.) injection of ~18 MBq of ^18^F‐NaF and were imaged 1 h later for 20 min in an Inveon microPET/CT (Siemens preclinical, Knoxville TN). Approximately 24 h later, the mice received an i.v. injection of 18 MBq of ^18^F‐FDG and were imaged 40 min later for 20 min via PET. At the end of the scan and based on the pHe studies where pHe began decreasing after 1 h, the mice received an i.v. injection of 25 mg/kg (0.6 mg/0.05 mL saline) of NSPS every 60 ± 15 min four times for a total treatment dose of 100 mg/kg (2.4 mg) NSPS in 0.2 mL solution (*n* = 4), which we refer to as one‐time 100 mg/kg NSPS, or vehicle (0.05 mL saline) every 1 h four times (*n* = 3). Within 18 h of the final NSPS/vehicle injection, the mice received ~18 MBq of ^18^F‐FDG again and were imaged 40 min later via PET for 20 min. The next day, the mice received ~18 MBq of ^18^F‐NaF and were imaged 60 min later via PET for 20 min. All data sets were reconstructed using the three‐dimensional (3D) ordered subset expectation maximization/maximum a posteriori (OSEM3D/MAP) algorithm into 128 × 128 × 95 slices with a voxel size of 0.095 × 0.095 × 0.08 cm^3^ at a beta value of 0.01. The PET images were normalized to the injected dose. Three‐dimensional regions of interest (ROIs) were drawn around the tumor and skeletal bone in the ^18^F‐NaF PET images and around the tumor, liver, heart, brain, kidneys, lungs, and muscle in the ^18^F‐FDG PET images. The radiotracer uptake in each ROI were compared between the post NSPS/veh administration and baseline. Based on the results, the ^18^F‐FDG PET scans were repeated 1 week later using the same protocol. Then the mice were euthanized and tumors were harvested. Paraformaldehyde‐fixed paraffin embedded sections were analyzed by immunohistochemistry for Ki 67 (proliferation) and cleaved caspase 3 (apoptosis).

The studies were repeated on MDA‐MB‐231 breast (*n* = 6 per group), 4 T1 breast (*n* = 5 per group), PC3 (*n* = 4 per group) prostate, HCA7 (*n* = 4 per group) colon, and H292 lung (*n* = 4 per group) tumor mouse models. The H292 mouse model was used as negative controls due to the absence of detectable TME‐HAP and absence of ^18^F‐NaF uptake in the tumor (please see results).

### 2.8 Tumor growth studies

We conducted a separate tumor growth study on 4 T1 tumor bearing mice that received either one‐time 100 mg/kg of NSPS (divided into 4 equal injections every ~1 h as described above) or saline (*n* = 10 per group). Tumor size was measured using calipers at 1 and 3 weeks post NSPS/saline injection.

### 2.9 Biodistribution of NSPS and toxicity studies

1. *Mass spectroscopy of tissue samples*. Balb/c mice bearing orthotopic 4 T1 breast tumors (~400 mm^3^) received i.v. injections of 25 mg/kg of NSPS or vehicle (saline). Then the mice were euthanized at the following time points (*n* = 3 per time point): 10 min, 1 and 4 h post NSPS administration. Tumor, blood, liver, spleen, bone, lung, heart, kidneys, brain, and muscle were harvested and flash frozen at −80°C. On the day of the analyses, the tissue samples were thawed and mixed with acetonitrile (1:1) and homogenized then centrifuged. The supernatants were extracted and subjected to mass spectroscopy in negative ion mode using the same setup parameters used to study the properties of NSPS described above.
2. *Bone toxicity studies*. Bone mineralization density was evaluated on the midshaft and metaphysis of femora, expected to be vulnerable to HAP depletion,^48^, ^49^ harvested from similar aged (12 weeks old) litter white female balb/c mice 10 days following i.v. treatment with either one‐time 100 mg/kg NSPS (*n* = 10) or vehicle (saline; *n* = 10). After aligning the long axis of the bone with the specimen tube, each femur mid‐diaphysis (1.848 mm in length) and the distal femur metaphysis (3.000 mm in length) were imaged using the following scan parameters (Scanco Medical μCT50 scanner, Brüttisellen, Switzerland): X‐ray tube voltage of 70 kVp drawing 114 μA, an isotropic voxel size of 6 μm, and an acquisition of 2000 projections per 360° rotation with an integration time of 300 ms. Weekly HAP phantom scans enabled conversion of attenuation values to bone mineral density following reconstruction. After contours were fit to the periosteal surface and endosteal surface (femur mid‐diaphysis) or inserted several voxels from the endosteal surface (distal femur metaphysis) per previously published studies,^50^, ^51^ we applied Scanco evaluation scripts to each region of interest to determine cortical bone and trabecular bone parameters. The global threshold was >828.1 mgHA/cm^3^ (Gaussian image noise filter: sigma = 0.2 and support = 1) and > 417.2 mgHA/cm^3^ (sigma = 0.8 and support = 1) for the mid‐diaphysis and metaphysis, respectively. These scripts provided the following: cortical tissue mineral density (Ct.TMD), cortical thickness (Ct.Th), polar moment of inertia (J), resistance to bending on minor axis (*C*_min_), cortical bone area (Ct.Ar), trabecular tissue mineral density (Tb.TMD), trabecular bone volume fraction (BV/TV), trabecular number (Tb.N), and trabecular thickness (Tb.Th). Cortical porosity (Ct.Po), pore number, pore thickness, and pore spacing were also determined by applying a global, inverse threshold of <1021.3 mgHA/cm3 (sigma = 0.3 and support = 1) and evaluating the segmented image with an algorithm similar to the one for trabecular bone.
3. *Plasma calcium and phosphate levels*. Female Balb/c mice bearing 4 T1 tumors ~500 mm^3^ in size, as measured by calipers, were treated with either one‐time 100 mg/kg NSPS or saline injected i.v. (*n* = 9 per group). Approximately twenty‐four hours later, the mice were euthanized and blood was immediately collected via cardiac puncture. The blood was centrifuged and calcium and phosphate level were measured in the plasma using colorimetric assay kits (Abcam); ab102505 for calcium and ab65622 kit for phosphate levels according to the manufacturer's protocol. Briefly, 10 μL plasma was diluted in 40 μL deionized water (dH_2_O) in 96 well plates. Then 60 μL of calcium buffer and 90 μL of colorimetric reagent were added to each well and allowed to incubate at room temperature for 5–10 min. The wells were then scanned in a Gen 5.0 microplate reader (BioTek Industries, VT) in absorbance mode with a 575 ± 5 nm filter. For phosphate concentration, 20 μL plasma was diluted in 2.98 mL dH_2_O. Then 0.2 mL of the diluted samples were placed in 96 wells and 30 μL of the phosphate reagent was added to each well and allowed to incubate at room temperature for 30 min. Then the plates were scanned in a microplate reader with an absorbance filter of 650 ± 5 nm filter.
4. *Onco‐nephrology evaluation*. Nephrotoxicity is a common complication of many chemotherapeutics.^52^, ^53^, ^54^ Therefore, we carried out onco‐nephrology to assess renal function as follows: 4 T1 bearing mice were treated with one‐time 100 mg/kg NSPS. ~37 MBq of ^99m^Tc‐MAG3 were injected i.v. in 4 T1 bearing mice and with SPECT imaging^55^ within 48 h after NSPS administration.

## 3 RESULTS

### 3.1 Properties of NSPS

**FIGURE 1:**
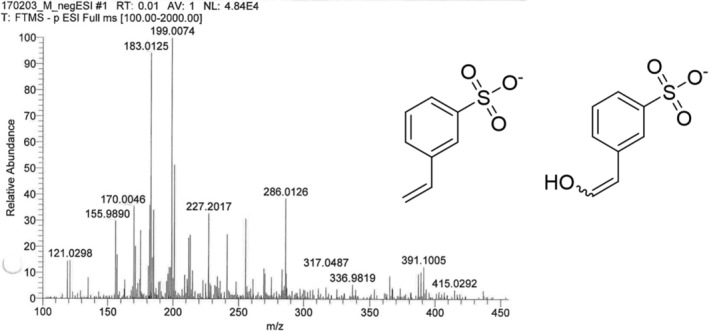
Predicted structures of major components of NSPS. Analytical data (mass spectroscopy) along with FT‐IR and NMR (shown in supplementary data) suggest vinyl benzenesulfonate (C_8_H_7_O_3_S^−^ [M‐H] exact mass 183.0125) and hydroxylated vinyl benzenesulfonate (C_8_H_7_O_4_S^−^ [M‐H] exact mass 199.0074) as predominant components of NSPS.

Triplicate independent analyses of NSPS samples resulted in the following findings:

- Fourier Transform Infrared Spectra (FT‐IR) and nuclear magnetic resonance (NMR) confirmed that NSPS was consistent with sulfonated polystyrene. Additionally, evaporation of water from the mixture yielded solutes with measured characteristics similar to Amberlite IR 120 Na^+^ beads and P‐NaSS (Ps‐100),^56^, ^57^ see supplementary Figures S1 & S2 and supplementary Table S1.
- Mass spectra were dominated by two novel singly charged ions of 183.0125 *m/z* and 199.0074 *m/z* in negative ionization mode (see Figure 1). These features are consistent with small NSPS fragments vinyl benzenesulfonate (C_8_H_7_O_3_S^−^) and hydroxylated vinyl benzenesulfonate (C_8_H_7_O_4_S^−^), respectively (see Figure 1). Few ions were detected in the range between 500 and 4000 *m/z*.
- The ultraviolet (UV) absorption spectrum showed increasing absorption between 300 and 200 nm consistent with scattering and absorption features at ~228 and ~ 255 nm typical of polystyrene^58^ (see supplementary Figure S3) which shares similar carbon backbone structure to the basic repeating unit of Amberlite AIR 120 Na^+^ and P‐NaSS (Ps‐100).
- Dynamic light scatter (DLS) of the filtered solution of NSPS showed the formulation to be nanoparticulate with a hydrodynamic diameter of 159.09 ± 6.39 nm and a poly dispersity of 0.22 ± 0.02 (see supplementary Figure S4) consistent with an amphipathic polymer that consists of particles with a relatively narrow size distribution. Based on the DLS measurements, we estimate the size of NSPS to be at least 56 kilodaltons (kDa) and up to 770 kDa, assuming spherical structure (see detailed calculations in supplementary data).

In conclusion, NSPS formulation is a stable nanoparticulate that is soluble in aqueous media. It consists of associated monomers with a distribution of sizes. Regarding storage, NSPS should be refrigerated. Annual mass spectrometer tests of our formulation revealed that NSPS maintained its structural properties 1 year after formulation. No convincing evidence of masses 199 and 182 Da were found 2 years after formulation (see supplementary Figure S5).

**FIGURE 2:**
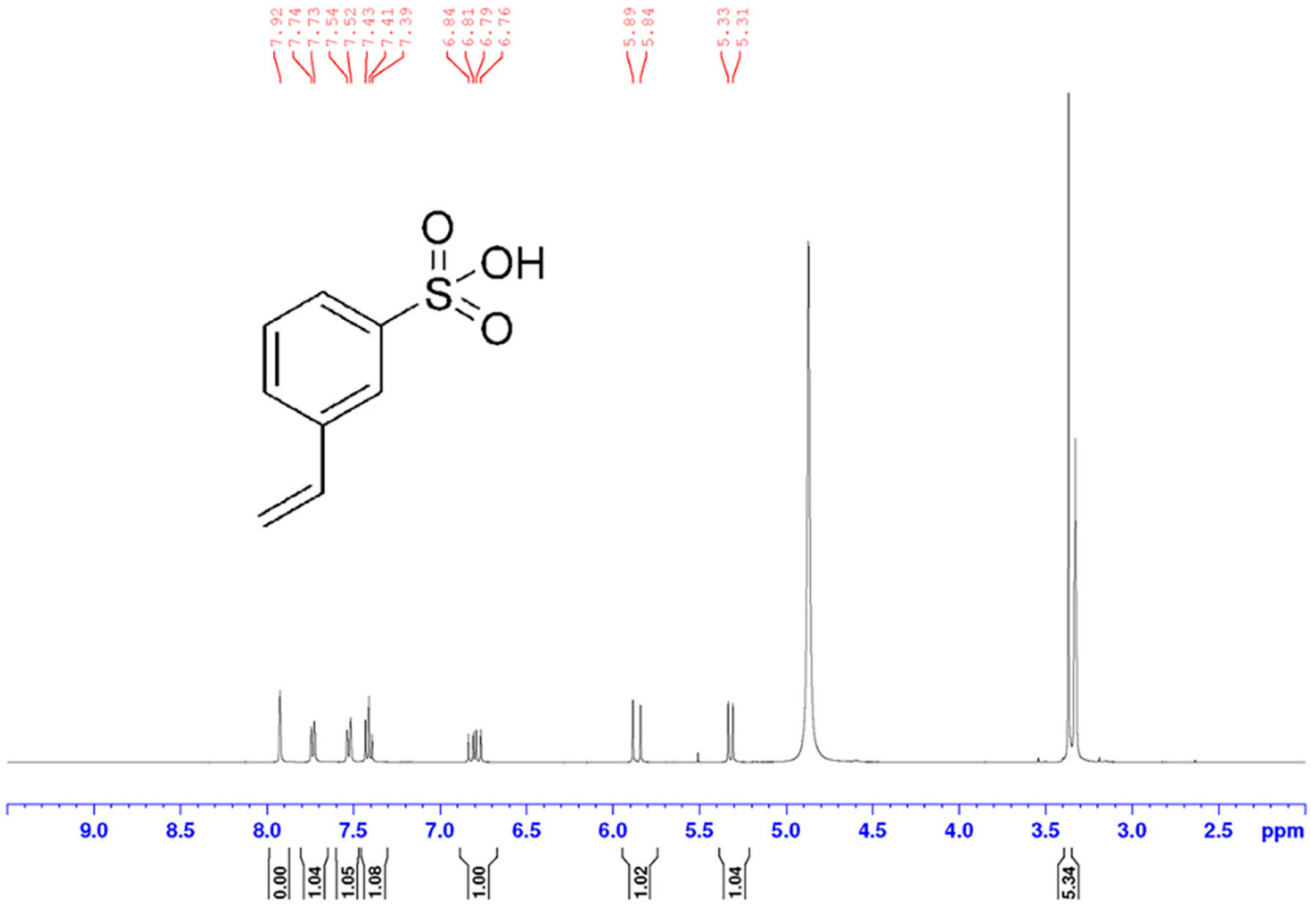
Structure of the novel NSPS monomer. Structure and corresponding 1H NMR spectrum of the small molecule VU0945652 formulated at Vanderbilt University and postulated to be the active ingredient of NSPS.

To validate that the 199 Da monomer, which had not been known or found commercially, is the active ingredient in NSPS, we successfully synthesized a novel hydroxylated vinyl benzenesulfonate monomer of mass 199 Da (VU0945652) shown in Figure 2. Both the nanoparticulate and monomer were found to be efficacious in killing tumor cells containing extracellular HAP as described below.

### 3.2 NSPS increases pH in calcium phosphate phantoms

In the phantoms that contained 10 mL H_2_O and 1 mg calcium phosphate, pH increased in the presence of 1X NSPS (~4 nmol/mL for 55 kDa molecular weight) within 5 min and this increase persisted over (1 week); pH at baseline of 10 mL H_2_O with or without 1 mg of CaPO4 salt was 6.23 ± 0.05 (mean ± STD) and 6.41 ± 0.11 after adding NSPS to the 10 mL H_2_O only (*p* > 0.05) and significantly increased to 6.92 ± 0.11 (*p* < 0.05) after adding NSPS to the H_2_O + CaPO4 mix.

### 3.3 NSPS dependent cytotoxicity is co‐incident with cells lines containing microenvironmental HAP

**FIGURE 3:**
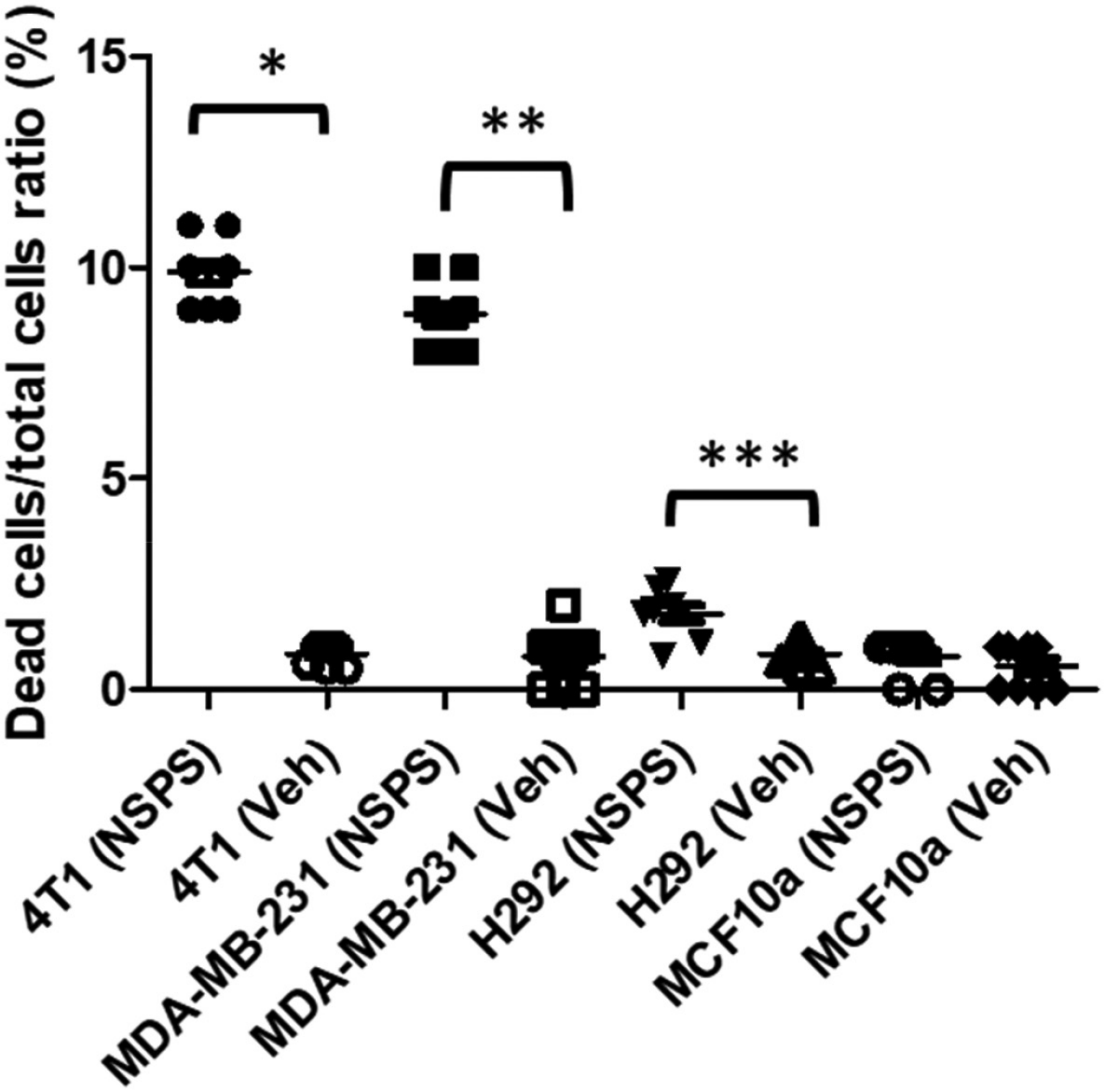
NSPS leads to tumor cell death in cells with HAP. Dead to total cell ratio of 4 T1, MDA‐MB‐231 tumorigenic breast cell lines (both deposit HAP in their extracellular matrix), H292 lung tumor cell line which had no detectable ECM‐HAP, and MCF10 normal breast cell line that lacks extracellular HAP. All cells treated with 7 μM NSPS and counted within 18–24 h. * *p* < 0.0001; *t* = 33.2, df = 16; ** *p* < 0.0001; *t* = 23.7, df = 16; *** *p* = 0.0003, *t* = 4.6, df = 16; MCF10a; *p* = 0.3464; *t* = 0.97, df = 16.

**FIGURE 4:**
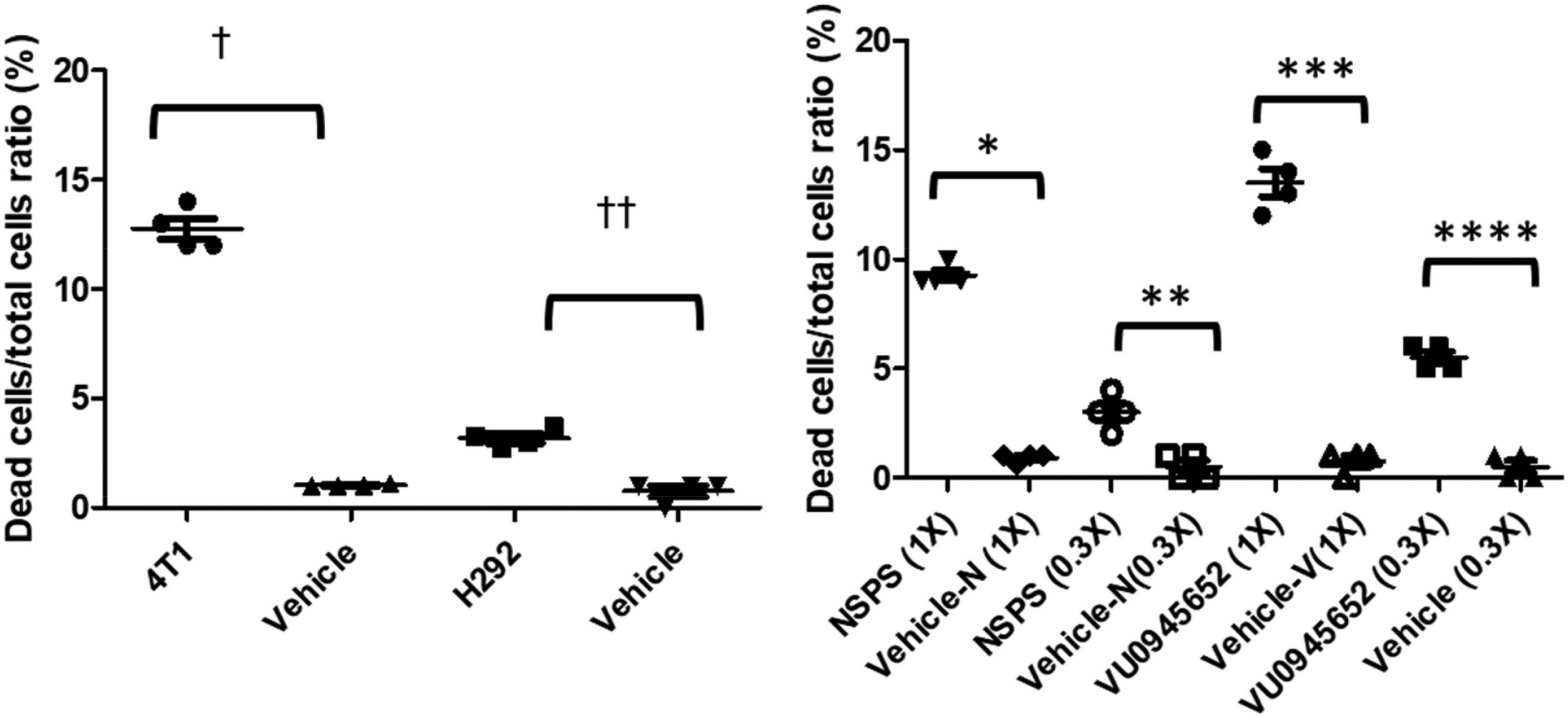
VU0945652 is the active ingredient of NSPS polymer and tumor cell death is dose dependent. (Left) Impact of 20 mg of VU0945652 on 4 T1 breast and H292 lung tumor cultures. (Right) Dose effects of NSPS polymer (1X = 2.4 mg) and the small monomer VU0945652 (1X = 20 mg) on 4 T1 breast cells cultured in osteogenic cocktail medium. Controls are cell cultures that received saline with culture media. ^†^ *p* < 0.0001; *t* = 22.22, df = 6; ^††^ *p* < 0.0001; *t* = 9.99, df = 6; * *p* < 0.0001; *t* = 31.01, df = 6; ** *p* = 0.0025; *t* = 5.000, df = 6; *** *p* < 0.0001; *t* = 18.42, df = 6; **** *p* < 0.0001; *t* = 12.25, df = 6.

Within 24 h of adding the small concentration of 1X of NSPS (~7 μM), we observed significant tumor cell death (~ 10%, *p* < 0.05) in 4 T1 and MDA‐MB‐231 cultures that contain TME‐HAP.^11^, ^12^ NSPS had minimal impact (minimal tumor cell death) on H292 cell cultures that lack significant detectable ECM‐HAP (see Figure S6) while NSPS had no impact on the MCF10a normal breast cells with cell death comparable to those receiving vehicle (*p* < 0.05), see Figure 3. We attribute the lack of response to NSPS to the absence of ECM‐HAP in the MCF10a cells.^3^ On the other hand, the small but significant effect of NSPS on H292 cells may suggest the presence of low levels of ECM‐HAP in that cell line that is not revealed by our staining protocols (see supplementary data). For the 4 T1 cell line, we observed dose dependent cell death when we tested varying (1X and 0.3X) concentrations of NSPS (see Figure 4). We obtained greater efficacy on the tumor cells with ~16 μmol/mL of VU0945652 (see Figure 4). This high dosage of the small molecule VU0945652 is expected as at least 2 of the monomers would have to reach a HAP lattice simultaneously to chelate calcium as described above. However, the study demonstrates that VU0945652 is the active cytotoxic ingredient of NSPS polymer and we are conducting ongoing studies to expand VU0945652 to a bridged multimer with exact known masses and shape.

### 3.4 NSPS temporarily increases pHe in tumors with TME‐HAP, in vivo

**FIGURE 5:**
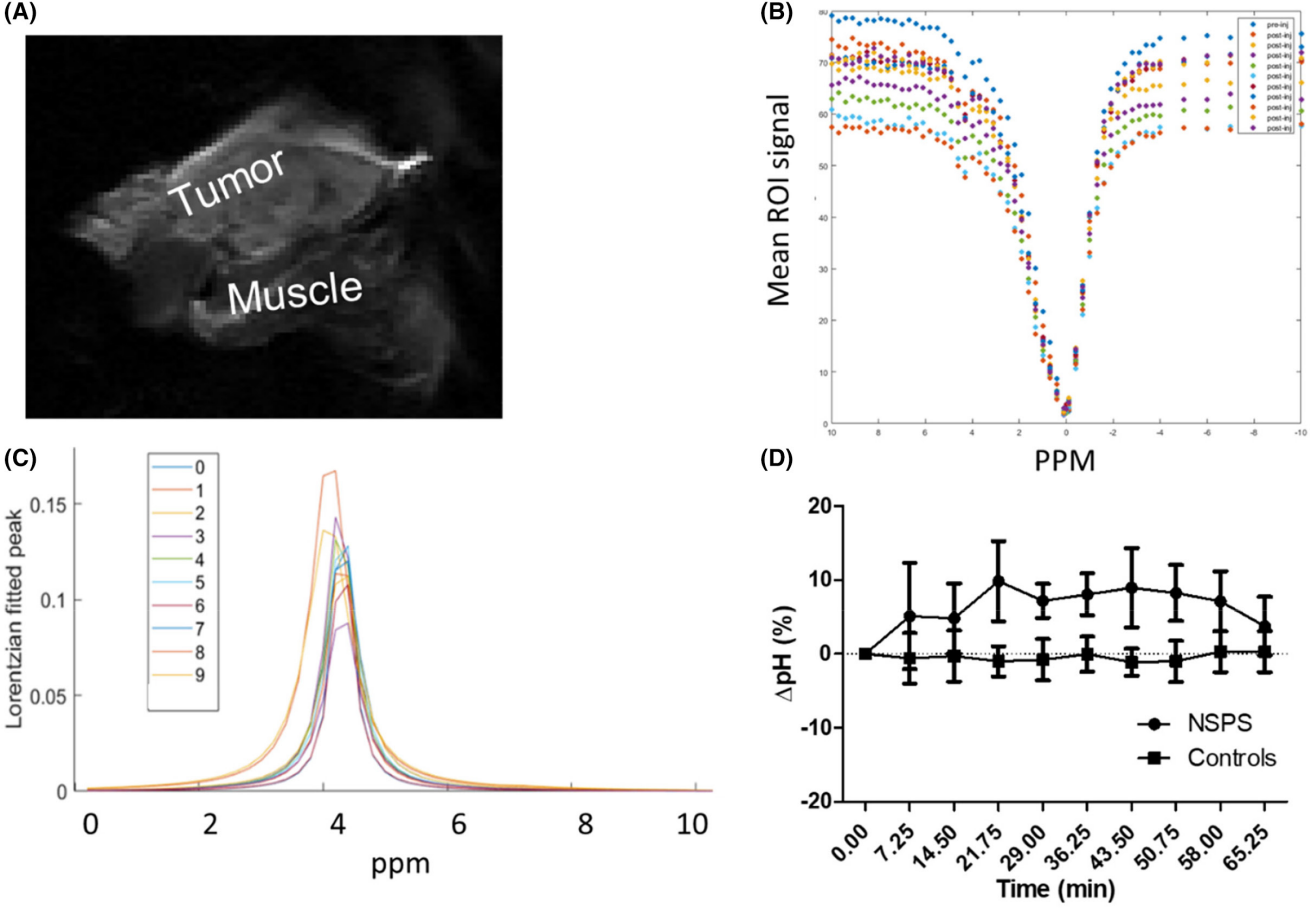
NSPS induces acute alkalosis localized to the tumor. Panels A‐C are typical data from a single NSPS‐treated mouse bearing a 4 T1 tumor in the mammary fat pad. (A) Sample T2 weighed MRI spectrum of a 4 T1 breast tumor in the mammary fat pad of a white female Balb/c mouse. Regions of interest were drawn around the tumor. (B) CEST data acquired after injection of iohexol in the tumor (baseline or scan 0), just after i.v. injection of 25 mg/kg NSPS and every 7.25 min for a total of 10 scans post NSPS treatment. (C) Lorentzian fit of the tumor CEST data around the 4.3 ppm peak. (D) Changes in tumor extracellular pH compared to baseline (scan 0) is the average of 4 independent measurements per group (NSPS vs vehicle saline injected controls) on 4 T1 tumor bearing mice. Data points displayed as means ± SE.

Using CEST MRI, we found extracellular pH (pHe) in the 4 T1 breast tumors, located in the mammary fat pad, to increase by nearly 10% from baseline before gradually receding back to baseline during the first hour post NSPS administration, see Figure 5. As expected, no changes in pHe were detected in the muscle of the NSPS‐treated mice, in 4 T1 tumors of mice receiving vehicle (controls), or in H292 tumors receiving NSPS due to the absence of TME‐HAP in the H292 tumors (see supplementary Figure S7).

**FIGURE 6:**
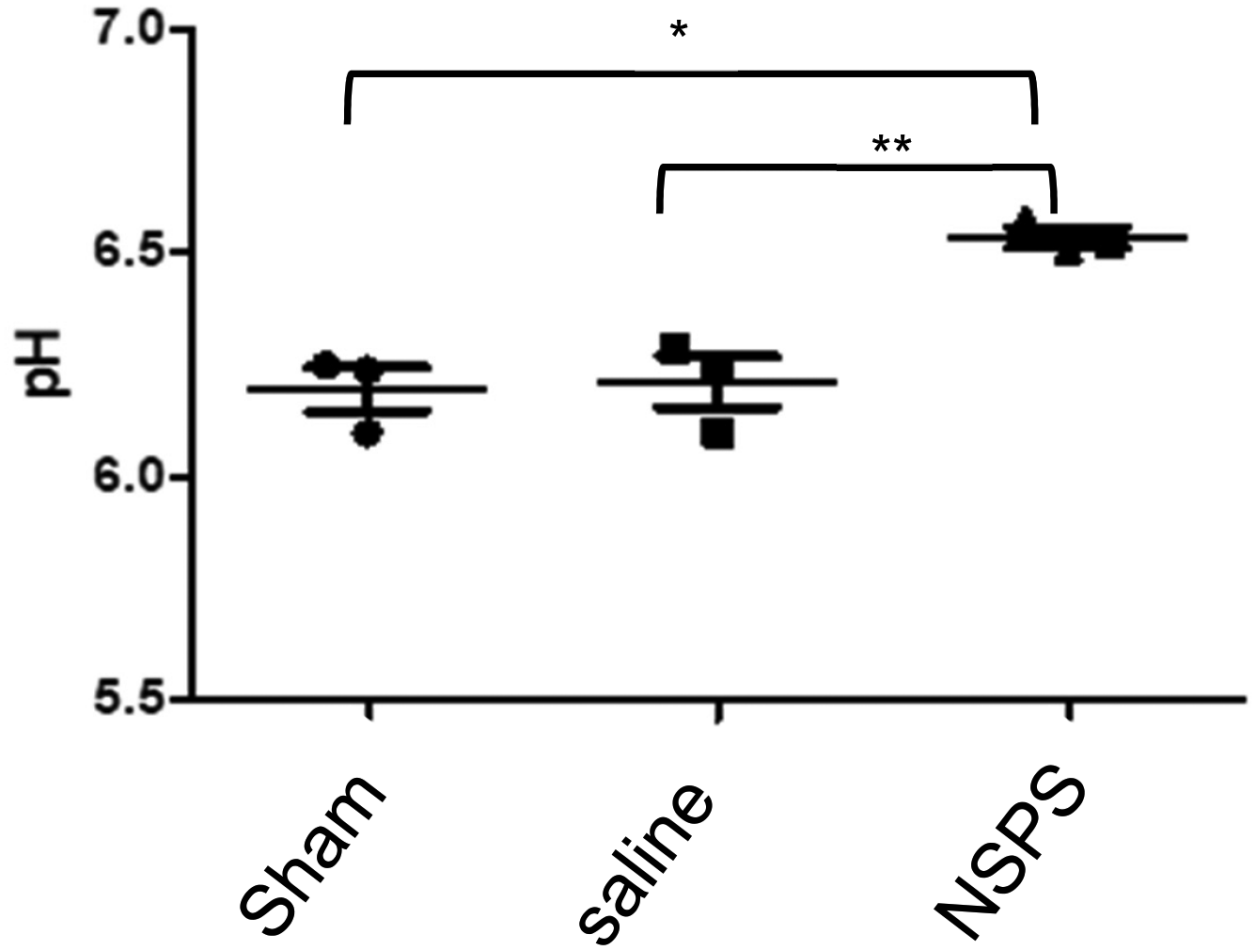
NSPS increases tumor pH in vivo. Groups of mice bearing 4 T1 tumors in the mammary fat pad received 25 mg/kg NSPS (i.v.), vehicle (saline), or nothing at all (shams); *n* = 3 per cohort. The tumors were then homogenized and the overall (macro) pH of tumors that received NSPS was significantly higher (*p* < 0.05) than that of the other two groups. * *p* = 0.0031, *t* = 6.40, df = 4; ** *p* = 0.0064, *t* = 5.23, df = 4; sham vs saline: *p* = 0.8359, *t* = 0.22, df = 4.

The pH in the ex vivo tumor homogenates of the NSPS‐treated mice was 6.51 ± 0.04 (mean ± SD) and significantly higher (*p* < 0.05) than the pH of the tumors from the vehicle treated mice (pH = 6.19 ± 0.1) or from untreated (sham) mice (pH = 6.20 ± 0.08) (see Figure 6). The pH of the homogenates is a measure of the average acidity that includes the extra and intracellular contents of the tumors and does not take into account tumor and extracellular heterogeneity, that is, this study measured the macro pH of the tumor. Thus, the change in micro extracellular pH (i.e., the decreased acidity of the extracellular space) after NSPS treatment, is expected to be much larger than is observed in the tissue homogenates. Taken together, the in vivo pHe and ex vivo tumor pH results strongly suggest the acute alkalosis due to NSPS treatment.

### 3.5 In vivo efficacy of NSPS

We originally hypothesized that achieving tumor efficacy, in vivo, through dissolution of TME‐HAP would be a two‐step process: (i) create an injectable drug that could chelate one of the components of HAP, which in our case is NSPS to chelate calcium; and (ii) rapidly dissolve as much TME‐HAP as possible to maintain a localized alkalosis status long enough to have a significant impact on the tumor without affecting normal cells. Based on the pHe results, we initially tested a maximum practical dose of 2.4 mg NSPS dissolved in 0.2 mL saline. This is the equivalent of nearly 100 mg/kg NSPS for an average mouse weight of about 25 g. To take advantage of the time it takes pHe to reset back to normal levels (~1 h), we initially tested the following dosing regimen protocol: tumor bearing mice were treated with 25 mg/kg/0.05 mL every ~1 h up to a total of 100 mg/kg/0.2 mL. A quarter of the NSPS that we made was injected intravenously (tail vein, jugular catheter, or retro‐orbital) every hour 4 times till the entire 2.4 mg/0.2 mL were administered. This dosing regimen has proven to be highly efficacious in treating tumors containing TME‐HAP with minimal adverse effects as we have shown below. Therefore, we continued with that dosing regimen and we will refer to this dosing regimen as one‐time indicating that the 100 mg/kg of NSPS were administered in 1 day in under 6 h.

**FIGURE 7:**
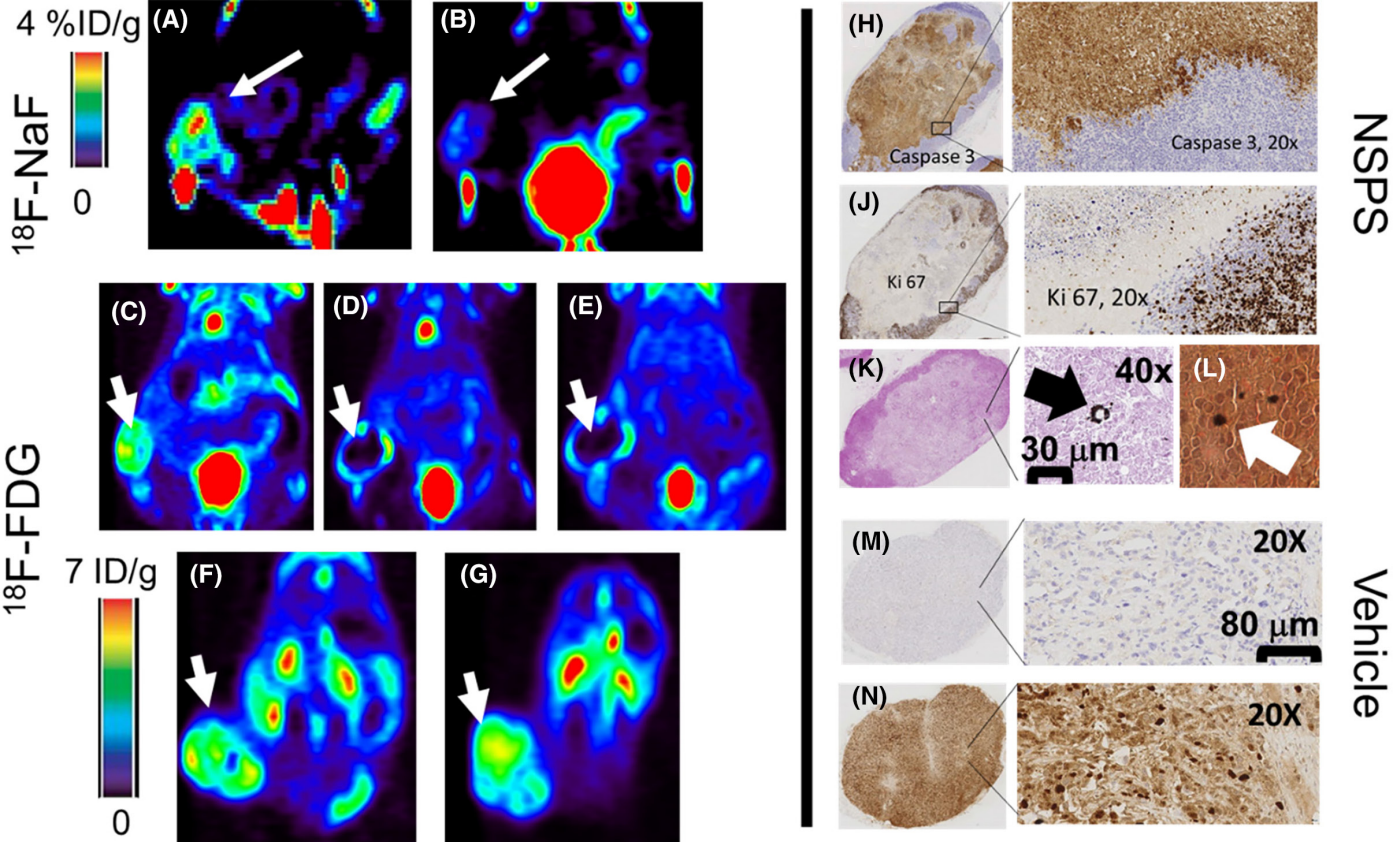
NSPS dissolves TME‐HAP and inhibits glucose metabolism for up to 1 week. Representative images of a MMTV‐Neu breast tumors in the mammary fat pad of female FVP/n mice imaged with ^18^F‐NaF at (A) baseline (white arrow points to tumor) and (B) within 48 h following i.v. injection of one‐time 100 mg/kg of NSPS (see Table 1 for quantification and statistics). Approximately 24 h following baseline ^18^F‐NaF imaging, the mice were imaged with ^18^F‐FDG PET at (C) baseline, (D) 24 h post NSPS treatment and (E) 1 week post NSPS treatment. (F) Same mouse model imaged with FDG PET at baseline and (G) 1 week post vehicle (saline) injections. All tumors from all mice were harvested after the 1‐week FDG scan and underwent IHC analyses. The tumors of the NSPS‐treated mice tested positive for cleaved caspase 3 (apoptosis) (H) throughout the tumors and negative for Ki 67 (proliferation) (J). Some HAP was observed in the tumor via von Kossa (K) and alizarin red S (L) staining. Black arrows point to positive (black) stains. This is consistent with ^18^F‐NaF uptake in the tumor following treatment with NSPS. The vehicle treated mice were completely negative for cleaved caspase 3 (M) and positive for Ki 67 (N).

In vivo NSPS dependent changes in mineralization were evaluated using ^18^F‐NaF. There was a significant reduction (*p* < 0.05) in ^18^F‐NaF uptake post NSPS treatment as expected; ^18^F^−^ uptake in the tumor = 3.8 ± 0.5%ID/g (percent of the injected dose per gram) at baseline compared to 1.8 ± 0.5 %ID/g following one‐time treatment with 100 mg/kg NSPS (compare Figure 7A,B). These results are concordant with dissolution of at least 50% of TME‐HAP by NSPS treatment. Note that because bone uptake of ^18^F‐NaF was ~9 times higher than tumor HAP uptake due to the large skeletal surface area, the bone signal appears saturated in the ^18^F‐NaF PET images as discussed in previous work.^11^, ^12^ ^18^F‐NaF uptake by HAP on skeletal bone surface appeared unaffected by NSPS treatment. ^18^F‐NaF uptake in skeletal bone was 30.2 ± 2.6 %ID/g pre‐NSPS and 29.7 ± 2.4 %ID/g post NSPS (*p* > 0.05). *Of similar importance, is that* ^*18*^ *F‐FDG uptake in the tumors was reduced by more than 75% compared to baseline within 24 h of treatment with one‐time NSPS* (compare Figure 7C,D and see Table 1 for quantitation) for the MMTV‐Neu mice, which persisted for at least 1 week (see Figure 7E). These results indicate significantly reduced cellular glucose metabolic activity in the tumors after treatment with NSPS. No changes were found in the radiotracers’ uptake in the tumors of the control groups that received saline (see Figures 7F,G and Table 1). Harvested tumors following the 1‐week ^18^F‐FDG scans revealed apoptosis (via cleaved caspase 3 staining, ~75%) throughout the tumors of the NSPS‐treated mice with minimal proliferation (via Ki 67 staining, <25%), see Figures 7H,J, respectively, indicating tumor cell death. No clear apoptosis signaling was found for the vehicle treated mice (see Figure 7K,L). On the other hand, ^18^F‐NaF uptake in the skeletal bone was 30.2 ± 2.6 %ID/g at baseline and 30.1 ± 2.4 %ID/g post NSPS (*p* > 0.05) or vehicle (i.e., no noticeable changes in bone HAP). We present more results of toxicity studies below.

**TABLE 1:** ^18^F‐FDG concentrations (%ID/g) in the MMTV‐Neu orthotopic model at baseline and post treatment with either 100 mg/kg/0.2 mL of NSPS one‐time or vehicle (0.2 mL saline one‐time).

Time	^18^F‐FDG concentration (%ID/g)						
	Tumor	Liver	Brain	Myocardium	Lungs	Kidneys	Muscle
Baseline	3.8 ± 0.6 (*n* = 7)	1.3 ± 0.2	6.1 ± 0.5	5.1 ± 0.9	1.0 ± 0.3	4.2 ± 0.9	0.9 ± 0.3
Post NSPS	0.8 ± 0.5 (*n* = 4) (*p* = 0.0098)	1.6 ± 0.3 *p* > 0.05	5.7 ± 0.6 *p* > 0.05	4.9 ± 0.9 *p* > 0.05	1.2 ± 0.3 *p* > 0.05	3.3 ± 0.5 *p* > 0.05	0.6 ± 0.2 *p* > 0.05
Post Vehicle	3.6 ± 0.6 (*n* = 3) (*p* > 0.05)	1.3 ± 0.2 *p* > 0.05	6.0 ± 0.5 *p* > 0.05	5.3 ± 0.9 *p* > 0.05	1.0 ± 0.3 *p* > 0.05	3.5 ± 0.6 *p* > 0.05	1.0 ± 0.3 *p* > 0.05

*Note*: Numbers displayed as mean ± SD. *p* values are for the differences in FDG uptake between baseline and post NSPS or vehicle administration in each organ.

We obtained similar results to those of the MMTV‐Neu tumor mice in other cancer models including the MDA‐MB‐231 breast, 4 T1 breast, PC3 prostate, and HCA‐7 colon tumors (see supplementary Figure S8 and Table S2). All tumors were associated with TME‐HAP as indicated using alizarin red S & von Kossa staining (see e.g., supplementary Figure S8). Following one‐time treatment with 100 mg/kg NSPS, the MDA‐MB‐231 were imaged 1 week later with FDG while the rest of the mouse models were imaged with FDG at 24 h, then euthanized. IHC on the tumors revealed ~10% apoptosis (cleaved caspase 3) and ~ 90% Ki 67 at 24 h post NSPS treatment (see supplementary Figure S8).

**FIGURE 8:**
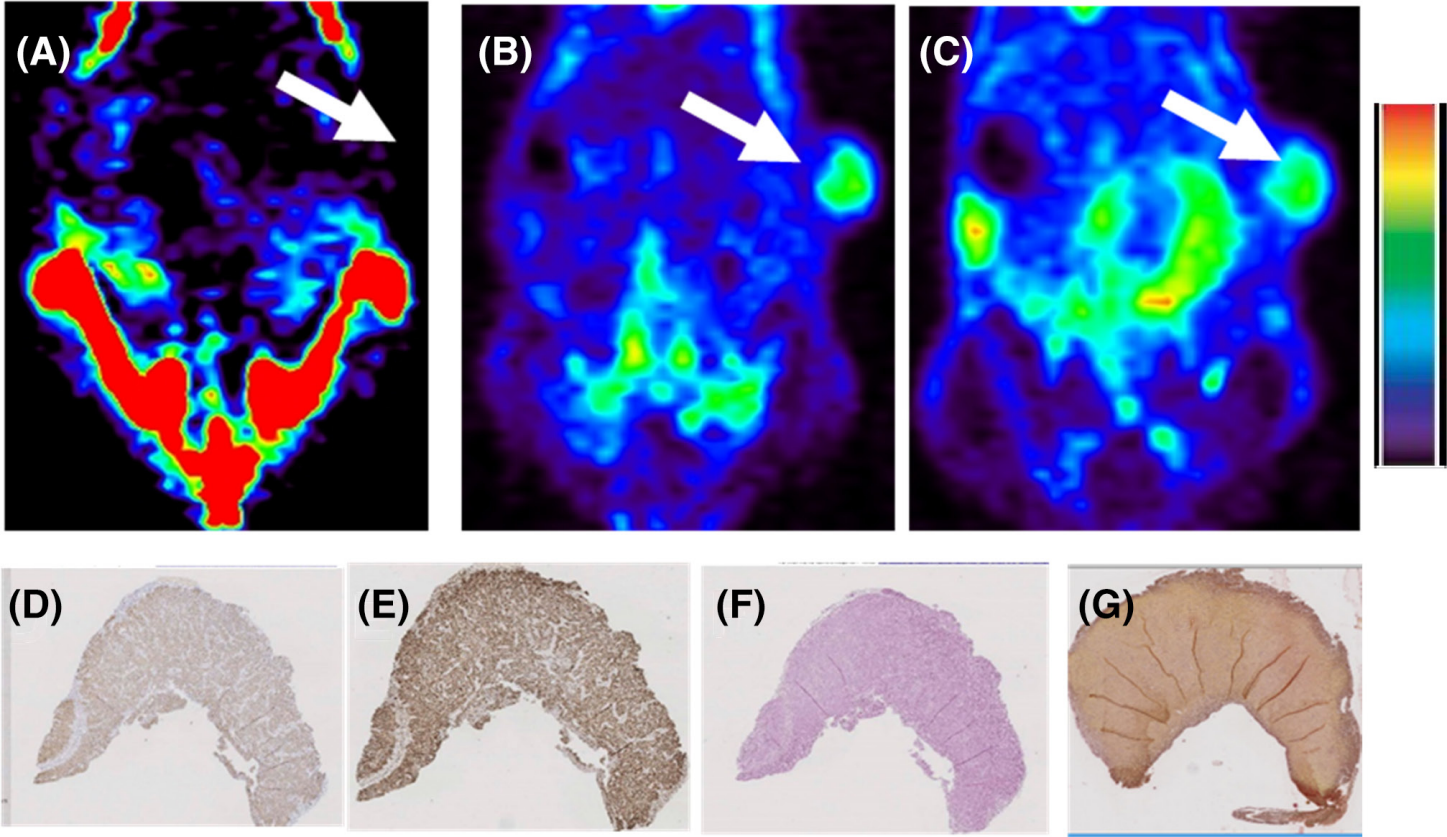
NSPS has minimal impact on tumors lacking detectable extracellular HAP. Representative image of a xenograft model of H292 lung tumor imaged with (A) ^18^F‐NaF PET but the tumor was not detected with this radiotracer. White arrows point to where the tumor should be. (B) Same mouse imaged with ^18^F‐FDG PET at baseline and (C) 24 h post treatment with 100 mg/kg NSPS. No changes in FDG uptake in the tumor were detected; ^18^F‐FDG uptake in the tumor at baseline and post NSPS was 2.3 ± 0.5 %ID/g in both cases. (D) Sections of harvested tumors which did not reveal positive cleaved caspase 3 but were positive for Ki 67 throughout the tumor (E). The tumors also tested negative for (F) von Kossa and (G) alizarin red S staining indicating absence of detectable TME‐HAP. Thus, this H292 mouse model serves as an in vivo negative control for testing NSPS.

While the H292 tumors were not detected with ^18^F‐NaF PET (see Figure 8A), they were naturally metabolically active and, therefore, easily detected with ^18^F‐FDG PET (see Figure 8B). One‐time100 mg/kg NSPS had minimal effect on the subsequent uptake of ^18^F‐FDG (3.1 ± 0.4 %ID/g *p* > 0.05, see Figure 8C). Additionally, IHC demonstrated no evidence of tumor cell death (see Figure 8D,E for cleaved caspase 3 and Ki 67 staining, respectively). These results are consistent with the absence of detectable TME‐HAP in these tumors as revealed by the absence of significant von Kossa and alizarin red S staining in these tumors, see Figure 8F,G, respectively.

### 3.6 Tumor growth studies

**FIGURE 9:**
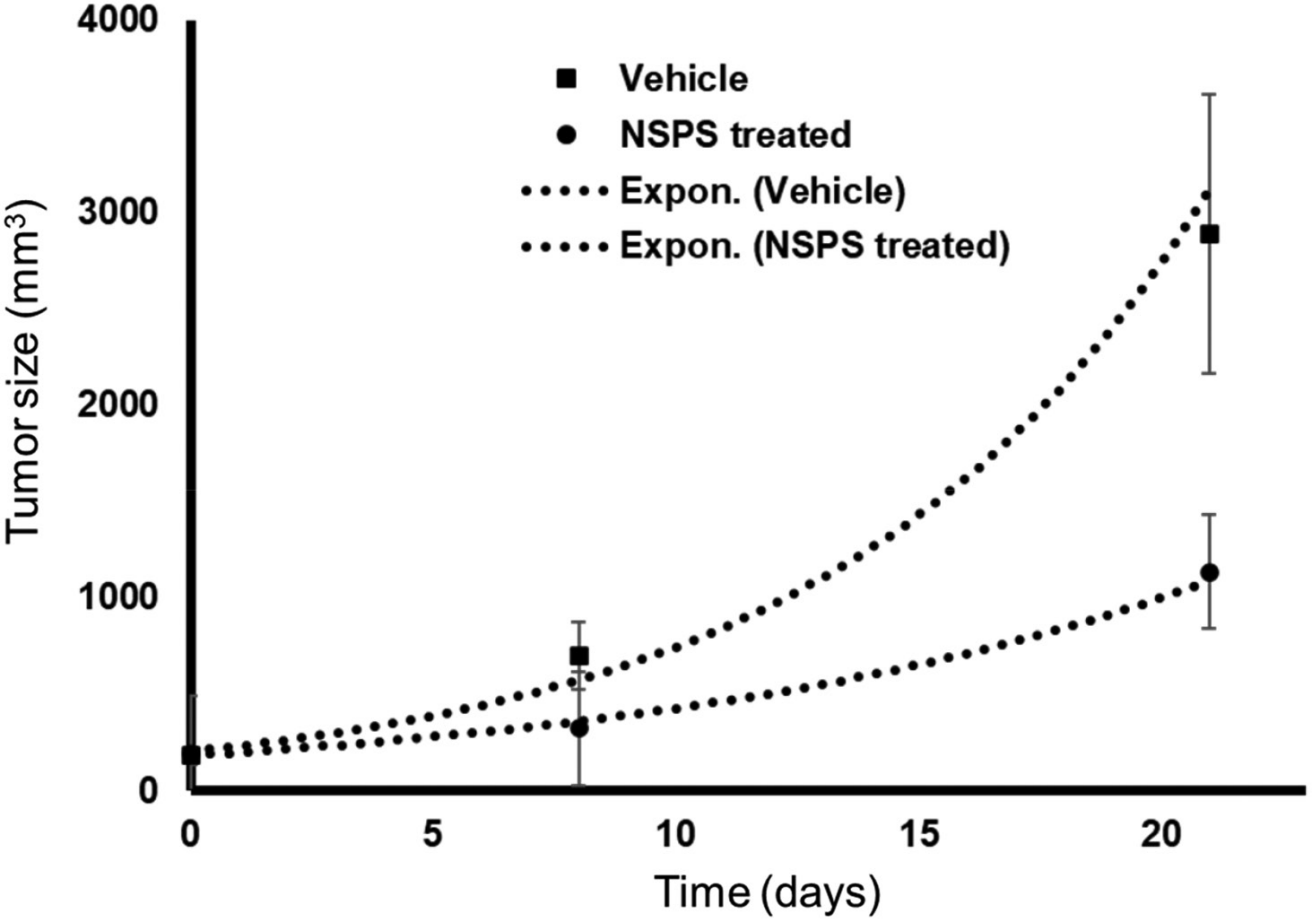
One‐time NSPS significantly inhibits tumor growth rate (tumor indolence). Growth curves of MDA‐MB‐231 xenograft breast tumors in female athymic nu/nu mice. Data were fitted with exponential growth curves (dashed lines). See text for fit parameters.

Tumor growth was significantly slower (*p* < 0.05) in the mice treated with one‐time NSPS (see Figure 9). Using the standard exponential growth model in GraphPad Prism (v 5.0); Y = Y_o_.e^kX^ where Y is tumor size, X is time in days and k is growth rate constant, the tumor size doubling time was measured to be 7.1 and 5.7 days for the NSPS‐treated vs control mice, respectively. The treatment/control (T/C) tumor size ratios^59^ at day 21 = 39% ± 10% or ~ 40% reduction in tumor growth rate following one‐time treatment with NSPS. We attribute the reduction of tumor growth rate by NSPS to impacts on tumor metabolic activity and increased tumor cell death following treatment.

### 3.7 Biodistribution and toxicity studies

**FIGURE 10:**
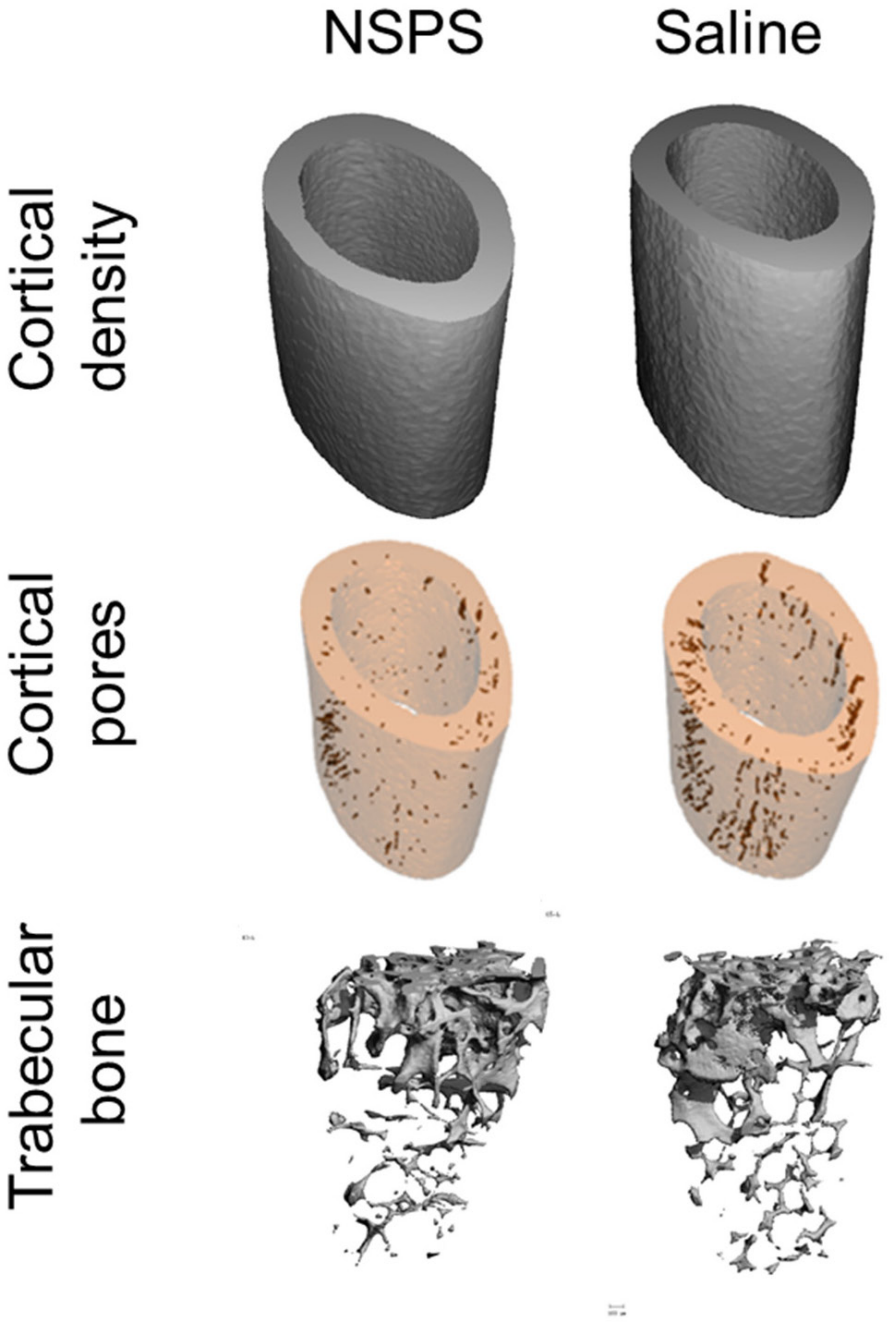
NSPS has limited impact on skeletal bone. Three‐dimensional microCT images of femur bones harvested from age‐matched white balb/c mice. Red streaks on bone are the pores. The mice received either 100 mg/kg one‐time NSPS (left) or vehicle saline (right) 10 days before harvesting. The bone specimen were imaged in a microCT at a nominal resolution of 6 μm.

**FIGURE 11:**
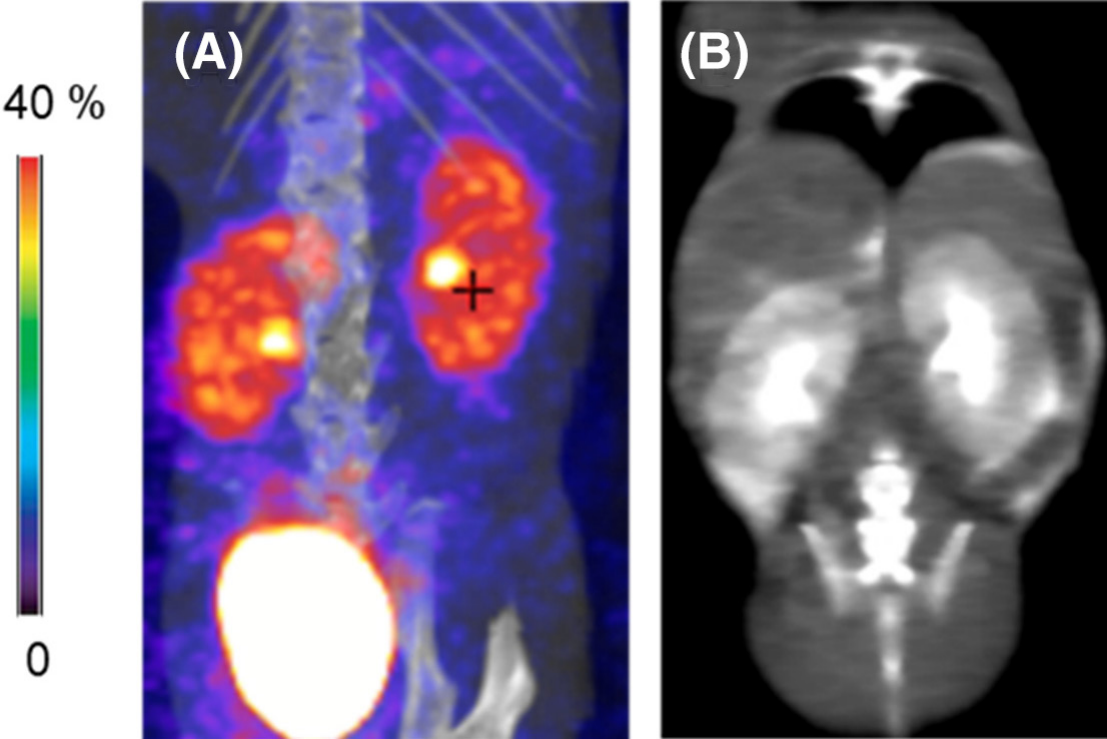
No evidence of nephrotoxicity following treatment with NSPS. Mice imaged with (A) ^99m^Tc‐MAG3 SPECT (for renal function) and (B) with CT following an i.p. injection of Optiray CT contrast The images were taken 24–48 h after treatment with 100 mg/kg of NSPS. Normal renal function detected in both images.

1. *Mass spectrometry of tissue samples*. We note the following observations in this study: (i) NSPS monomers appear to be present without the hydroxyl group at a mass of 181.97 Da; (ii) Initial preliminary analysis detected NSPS signature in tumors, whole blood (but not plasma), bone and whole heart (perhaps attributable to whole blood content) at 10 min and 1 h (see supplementary Figure S9). However, NSPS was detected in the tumors only at 4 h but not in bone or whole heart. No significant NSPS signature was detected in the liver, spleen, muscle, kidneys, and brain extracts at any time. This lack of detection may be an issue of sensitivity of some of the tissue extracts. As expected, we found no NSPS signature for tumors from mice that received vehicle (see Figure S9F).
2. *Bone toxicity studies*. We found no significant differences (*p* > 0.05) in any of the bone microCT parameters measured across the midshaft (see Table 2 and Figure 10) or in the trabecular bone (see Table 3 and Figure 10). Thus, NSPS, even at 100 mg/kg, had minimal impact on bone mineralization and bone growth.
3. *Plasma calcium and phospate levels*. There were no significant differences in calcium or phosphates levels of the NSPS‐treated versus the control mice at 24 h; Ca^2++^ levels in plasma of NSPS‐treated and control mice were 0.43 ± 0.01 and 0.42 ± 0.02 μg (mean ± SE) (*p* = 0.620), respectively. Plasma phosphates levels were 2.89 ± 0.09 and 2.78 ± 0.28 mmol/L (*p* = 0.347), in the NSPS‐treated and controls, respectively. Cation exchange is a rapid and reversible process where cations like calcium are exchanged on an equivalent charge basis,^60^ that is, Na^++^ for Ca^++^ and vice versa. NSPS has the potential to temporarily alter local calcium equilibria but does not cause systemic calcium deficiency.
4. *Nephrotoxicity*. No signs of fibrosis or renal dysfunction were observed in the ^99m^Tc‐MAG3 SPECT studies (see Figure 11A). Additionally, contrast CT showed normal renal cortex and renal pelvis (see Figure 11B). Thus notably, there was no evident nephrotoxicity due to NSPS treatment, results consistent with the absence of evidence of HAP in the renal microenvironment.

**TABLE 2:** NSPS has minimal impact on bone mineralization.

Study	Ct.TMD (mg HA/cm^3^)	J (mm^4^)	C_min_ (mm^3^)	Ct. Ar (mm^2^)	Ct.Th (mm)	Ct. Po %	Pore number (1/mm)	Pore Thickness (mm)	Pore Space (mm)
NSPS	1269 ± 31	0.269 ± 0.022	0.152 ± 0.009	0.678 ± 0.047	0.160 ± 0.005	0.122 ± 0.082	2.643 ± 0.238	0.010 ± 0.003	0.400 ± 0.059
Vehicle	1262 ± 20	0.218 ± 0.043	0.130 ± 0.018	0.632 ± 0.080	0.152 ± 0.003	0.126 ± 0.089	2.563 ± 0.228	0.011 ± 0.004	0.409 ± 0.064

*Note*: Toxicity measurements on femur bone harvested from female white balb/c mice 10 days following injection of one‐time 100 mg/kg NSPS or vehicle (saline). Data presented as mean ± SD (*n* = 10 per group). See text for abbreviated definitions. *p* > 0.05 between the two groups for any measurement.

**TABLE 3:** NSPS has minimal impact on bone growth.

Study	BV/TV	Tb.N	Tb.Th (μm)	Tb.TMD (mg HA/cm^3^)
NSPS	0.099 ± 0.025	2.95 ± 0.32	46.60 ± 2.19	981 ± 12
Vehicle (saline)	0.101 ± 0.021	3.01 ± 0.22	50.76 ± 3.52	990 ± 15

*Note*: Toxicity measurements on distal femur (trabecular bone) harvested from female white balb/c mice 10 days following injection of one‐time 100 mg/kg NSPS or vehicle (saline). Data presented as mean ± SD (*n* = 10 per group). *p* > 0.05 between the two groups for any measurement.

## 4 DISCUSSION

Although NSPS originated from the parent compound Amberlite IR 120, it is a novel bridged nanoparticle suspension dominated by the hydroxylated vinyl benzenesulfonate monomer with a unique mass of 199 Da. We have produced this compound, VU0945652, in the lab and shown to be an active component of NSPS that functions to breakup HAP by chelating calcium (via wrap‐around method) from the HAP lattice. The abundance of the released (PO4)^−3^ and OH^−^ anions and/or the potential formation of NaOH in the TME induce an acute alkalosis localized only to the TME. This perturbation of tumor pHe is localized and not systemically (whole body) and has not previously been described.

We have shown that a flat 2.4 mg/0.2 mL of NSPS, or the equivalent of about 100 mg/kg, inhibits tumor glucose metabolism and growth. While the number of animals per tumor model was limited, based on the original formulation, this was the maximum practical daily dose. It is not uncommon to administer a flat dosage of nanoparticles rather than a dose calculated in mg/kg.^61^, ^62^, ^63^ A pilot study at 0.6 mg or the equivalent of ~25 mg/kg had a modest but not significant (*p* > 0.05) impact on tumor glucose metabolism as assessed using ^18^F‐FDG PET imaging. Generally, polymer‐based drugs are administered at high doses to achieve therapeutic efficacy due to the intrinsic physicochemical properties that may limit circulation time in the blood plasma and/or poor aqueous solubility which reduces bioavailability.^64^ On the other hand, small molecule drugs are distributed not only in tumor tissue, but are also widely distributed in all healthy tissues, which may result in adverse side effects. This is one of the advantages of polymeric macromolecular drug formulations (like NSPS) which are likely to spare healthy tissue from toxicity by preferentially accumulating within tumor microenvironment through the enhanced permeation and retention (EPR) effect that appears mediated by disorganized tumor vasculature and poor lymphatic drainage of tumors.^64^ The mass spectroscopy biodistribution results confirm the presence of NSPS in the tumor. Meanwhile, absence of EPR and large vasculature to bone surface may explain absence of adverse effects of NSPS to bone. We postulate that the compact structure of HAP on bone surface, large skeletal surface area, limited vascularity of bone surface, and continuous bone remodeling^65^, ^66^, ^67^ may contribute to the limited interaction of NSPS with bone. By contrast, tumor HAP appears diffusely scattered within the extracellular matrix,^11^, ^12^ which combined with the large and disorganized tumor vasculature, affording susceptibility to calcium chelation and dissolution. Thus, there is an advantage to having a large size HAP dissolving agent such that it does not penetrate bone and is specific targeting to TME‐HAP. Furthermore, though trabecular bone CT parameters (BV/TV, Tb.Th, Tb.N, and Tb.TMD, see Table 3) of the treated mice appeared slightly lower, no significant differences were found in the overall bone microCT data between mice treated with NSPS and vehicle suggesting that NSPS at 100 mg/kg had minimal adverse effects on bone.

While 10% cell death may appear modest, in vitro, for cells containing ECM‐HAP, in the context of treatment with nanoparticles, 10% efficacy within 18 h is relatively high. Polymer‐based drugs typically take days and higher concentrations, for example, mM, to achieve similar efficacy.^68^, ^69^, ^70^, ^71^, ^72^

There are FDA‐approved cation exchange sulfonated polystyrene such as Kayexalate with high affinity for potassium used for patients with hyperkalemia with dosage as high as 800 mg/kg.^73^ Kayexalate is a linear polymer and has negligible affinity for calcium. As expected, it had no therapeutic effects on our tumor models even at the maximum dose dissolved in 0.2 mL saline and administered once per hour at a rate of 0.05 mL each hour as we did with NSPS.

Dissolution of TME‐HAP with NSPS had no perturbatory impact on blood concentrations of calcium and phosphates. These results indicate that NSPS treatment did not cause systemic effects, for example, release of calcium or phosphate from bone as we found no evidence of significant interaction of NSPS with bone. Although a localized response was evident in the tumor microenvironment via the ^18^F‐NaF, ^18^F‐FDG, and IHC analyses, the released calcium and phosphate following dissolution of TME‐HAP may be too modest to cause a detectible change in the concentrations of those molecules in the blood. These ions may be absorbed back into the tumors cells from the microenvironment via transcellular and paracellular pathways,^74^ and/or they may be absorbed by bone remodeling.

Following treatment with 100 mg/kg of NSPS, tumor glucose metabolism was inhibited for at least 1 week which may indicate an irreversible change in the tumor metabolic activity. Tumor acidity is one of the hallmarks of cancer and likely contributes to metabolic reprogramming.^75^ Of similar importance is that metabolic activity of normal tissue was uninterrupted by NSPS making dissolution of TME‐HAP an attractive cancer therapeutic target that, until now, has not been investigated. Tumor microenvironment is generally more acidic than the surrounding normal tissue.^76^, ^77^, ^78^, ^79^, ^80^ A sudden and rapid switch from acidosis to alkalosis may be toxic to tumors.^28^, ^81^, ^82^, ^83^, ^84^, ^85^ Modulation of the formulation of NSPS to reduce the nanoparticle size may further enhance efficacy.

We could not obtain evidence of TME‐HAP with H292 although we detected a modest impact of NSPS and VU0945652 on H292 in culture. We postulate that this may indicate that TME‐HAP may be associated with this cell line but is undetectable by von Kossa and alizarin red S even at 40× magnification. However, these data may indicate that the threshold of HAP abundance in the extracellular matrix needed to perturb the tumor microenvironment with NSPS, may be very small.

While no formal behavioral studies were carried out, we observed no changes in the overall behavior, food, or water consumption in any of the NSPS‐treated mice. Further, none of the NSPS‐treated mice exhibited cell lysis syndrome even within mice bearing large tumors (e.g., PC3). Cell lysis can occur with current chemotherapeutics.^86^ While some increased apoptosis (~10% increase) and reduced Ki 67(~10% reduction) was detected in the tumors at 24 h post treatment with NSPS compared to controls (see supplementary Figure S8), apoptosis was detected throughout the tumors by 1 week post NSPS treatment (see Figure 7). ^18^F‐FDG uptake was reduced by >75% in the tumors with HAP within 24 h post NSPS treatment so we postulate that the significantly reduced glucose metabolism results in increased tumor cell apoptosis at 1 week and consequently continued diminished FDG uptake in the tumor and reduced tumor growth rate. Therefore, tumor cell death may be slow enough to avoid cell lysis syndrome. However, more studies are needed to investigate whether cell lysis syndrome can occur in the presence of a large number of tumors in a single mouse following treatment with NSPS.

Taken altogether, the studies described above focused on obtaining results of key efficacy metrics^87^ for a novel cancer therapeutic, NSPS. *The results provide compelling evidence that NSPS treatment is efficacious in tumors with extracellular HAP with minimal impact on tissues that do not have HAP such as normal soft tissue or tumors lacking HAP*. The advantages of our cancer treatment approach include: (i) *Ubiquity*: any tumor with TME‐HAP is most likely to respond well to NSPS regardless of the tumor type or stage; (ii) *Response time*: pharmacodynamic parameters such as inhibition of tumor glucose metabolism, reduced proliferation, and increased tumor cell apoptosis can be observed within 24 h after administration of NSPS verifying efficacy, and (iii) *Lack of systemic toxicity*: we found no evident NSPS toxicity in normal tissue or skeletal bone.

We anticipate enhanced efficacy of NSPS at lower pH and for tumors that are more sensitive to pHe changes. However, NSPS treatment has limited efficacy in tumors that lack detectable TME‐HAP, for example, H292 lung tumor cells, and that may also include tumor heterogeneity in which HAP is not distributed throughout the tumor. In the latter case, we would expect to detect inhibition of glucose metabolism in the parts of the tumor that contain TME‐HAP but not the parts lacking TME‐HAP. Cox et al. and *Wen* et al. have demonstrated that tumorigenic cells produce and deposit HAP in cell culture when the culture medium contained ascorbic acid and β‐glycerophosphate (βG).^3^, ^10^ The production of tumor‐associated HAP, in vivo, therefore, may be dependent on a number of variables including distance to vasculature, dietary factors (e.g., vitamins C & D), and calcium regulation and concentration in the blood. Additionally, Han et al. have demonstrated that repeated injections of nanoparticulate HAP (nHAP) exogenously can reach tumors and indirectly results in endocytosis and inhibition of tumor cells at large doses.^31^ Though not the same as TME‐HAP, it may be possible that pretreatment of patients with nHAP for a few days to deliver HAP to tumors that lack HAP, or increased HAP abundance for tumors already containing HAP, followed by NSPS to induce alkalosis localized to the tumor would potentially maximize tumor efficacy. The disadvantage of such a dual therapeutic approach is that nHAP will also reach normal healthy soft tissue. Nonetheless, this dual therapy approach would broaden the application of NSPS to tumors like H292 lung that lack detectable TME‐HAP. Other potential ways to maximize tumor efficacy would be to pretreat tumors with NSPS to inhibit tumor glucose metabolism followed by conventional chemo and/or immunotherapeutics (perhaps at reduced doses) and minimize adverse effects of those therapeutics.

It is important to distinguish between our approach using NSPS to dissolve TME‐HAP to induce an acute localized alkalosis in the tumor microenvironment from other work that aimed at neutralizing tumor microenvironment using methods with (i) systemic sequelae, for example, treating with bicarbonates, (ii) inhibition of specific receptors that are also present in normal soft tissue,^28^, ^81^, ^82^, ^83^, ^84^ or (iii) targeting intracellular metabolism so as to reduce the intracellular pH and viability of tumor cells but without discrimination from normal soft tissue.^85^ While other prior strategies had efficacy in reducing tumor growth, confounding systemic effects were also evident, for example, changes in whole body pH and metabolism and attendant severe adverse effects such as extreme fatigue, seizures, and irreversible normal tissue damage^28^, ^81^, ^82^, ^83^, ^84^, ^85^ limiting clinical potential. Therefore, our strategy is distinct and designed to target TME‐HAP to temporarily elevate tumor pH locally without changing whole body pH. As HAP is generally absent in normal soft tissue and there are no other known injectable compounds that can breakup TME‐HAP in vivo, NSPS would be a one‐of‐a‐kind and first in a class of novel cancer therapeutics. NSPS could also potentially be used in the treatment other diseases in which HAP is expressed in the extracellular matrix, for example, hepatic metastases^14^ in the clinic or for detecting chronic tuberculosis in mice.^88^

## 5 CONCLUSION

We have shown that one‐time treatment with NSPS was efficacious in treating tumors with TME‐HAP as measured by reduced TME‐HAP mineralization, abrogation of tumor metabolic activity, and inhibition of tumor growth with minimal adverse effects. Attacking solid tumors by altering the pH of the TME only and not systemically by dissolution of TME‐HAP using NSPS is a novel approach to therapy. Dissolution of TME‐HAP using NSPS to elevate tumor pHe locally without changing whole body pH is an attractive approach to anticancer therapy. NSPS has significant potential to be a paradigm changing approach to the treatment of cancer patients with poor prognosis.

## AUTHOR CONTRIBUTIONS

**Mohammed N. Tantawy:** Conceptualization (lead); data curation (lead); formal analysis (lead); funding acquisition (lead); investigation (lead); methodology (lead); project administration (lead); resources (lead); software (lead); supervision (lead); validation (lead); visualization (lead); writing – original draft (lead); writing – review and editing (lead). **J. Oliver McIntyre:** Investigation (supporting); methodology (supporting); validation (supporting); writing – original draft (supporting). **Fiona Yull:** Visualization (supporting); writing – review and editing (supporting). **M. Wade Calcutt:** Formal analysis (supporting); writing – review and editing (supporting). **Dmitry S. Koktysh:** Formal analysis (lead); writing – review and editing (supporting). **Andrew J. Wilson:** Funding acquisition (supporting); writing – review and editing (supporting). **Zhongliang Zu:** Formal analysis (lead); methodology (supporting); writing – review and editing (equal). **Jeff Nyman:** Formal analysis (supporting); methodology (lead); writing – review and editing (lead). **Julie Rhoades:** Methodology (supporting); writing – review and editing (supporting). **Todd E. Peterson:** Methodology (supporting); writing – review and editing (equal). **Daniel Colvin:** Methodology (supporting); writing – review and editing (supporting). **Lisa J. McCawley:** Data curation (supporting); investigation (supporting); methodology (supporting); writing – review and editing (lead). **Jerri. M. Rook:** Investigation (supporting); methodology (supporting); writing – review and editing (supporting). **Barbara Fingleton:** Methodology (supporting); writing – review and editing (supporting). **Marta Ann Crispens:** Data curation (supporting); funding acquisition (supporting); investigation (supporting); writing – review and editing (lead). **Ronald D. Alvarez:** Funding acquisition (supporting); methodology (supporting); writing – review and editing (supporting). **John C. Gore:** Data curation (supporting); investigation (supporting); methodology (supporting); writing – original draft (supporting); writing – review and editing (equal).

## Supporting information


Data S1.

Data S2.

Data S3.

## Data Availability

The data that support the findings of this study are available from the corresponding author upon reasonable request.
