# Supplementary material for: Tumor therapy by targeting extracellular hydroxyapatite using novel drugs: A paradigm shift
Source: Cancer Med. 2024 Jan 18;13(3):e6812. doi: 10.1002/cam4.6812 (PMC11025459; doi:10.1002/cam4.6812)
Supplement: Supplementary file 1 — Data S1. [file CAM4-13-e6812-s002.docx]

# Supplementary data:

## Calculation or NSPS size.

To estimate the size of particles in a sample with a reported hydrodynamic diameter (d) of 159 nm and a polydispersity index (PDI) of 0.224, assuming a spherical shape and a density of 1 g/cm³, we can use the Stokes-Einstein equation:

d = k * (k_b * T) / (6 * pi * eta * r_h)

where d is the hydrodynamic diameter, k is the Boltzmann constant, k_b is the temperature, eta is the viscosity of the medium, and r_h is the hydrodynamic radius of the spherical particle.

Assuming room temperature (T = 298 K), the Boltzmann constant (k) is 1.38 x 10^-23 J/K, and the viscosity of water at room temperature is about 0.89 cP or 0.89 x 10^-3 Pa·s.

Rearranging the equation to solve for the hydrodynamic radius (r_h), we get:

r_h = k * (k_b * T) / (6 * pi * eta * d)

Substituting the given values, we get:

r_h = (1.38 x 10^-23 J/K) * (298 K) / (6 * pi * (0.89 x 10^-3 Pa·s) * (159 nm))

r_h = 7.74 x 10^-9 m or 7.74 nm

This calculation gives an estimated hydrodynamic radius of 7.74 nm for the spherical particles in the sample. However, it's important to note that this calculation assumes that the particles are perfectly spherical and have a density of 1 g/cm³, which may not be the case in practice. Additionally, other factors such as particle shape, surface charge, and interactions with the solvent can affect the hydrodynamic diameter measured by DLS, so additional characterization techniques may be necessary to confirm the size and properties of the particles.

For simplicity, we used the conversion tool provided by fluidic (<https://www.fluidic.com/toolkit/hydrodynamic-radius-converter>) which was designed for proteins. Our calculated hydrodynamic radius corresponds to 56 kDa for asymmetric geometry and 770 kDa for a spherical structure, by analogy to unfolded and folded proteins, respectively.

It is important to note that our calculations make several assumptions and simplifications, and the actual molecular weight of the particles may be different due to factors such as the shape and density of the particles, as well as the presence of any functional groups, e.g., sulfate.

## MRI acquisition protocol for acidoCEST studies.

Mice were anesthetized via inhalation of 2%/98% isoflurane/oxygen. Animals were secured in a supine position and placed in a 25-mm inner diameter quadrature radiofrequency (RF) coil (Doty Scientific, Columbia, SC). The RF coil and animal were then placed in a Bruker horizontal bore 7T imaging system (Bruker Biospin, Billerica, MA) for image acquisition. Respiration rate and internal body temperature were continuously monitored, and a constant body temperature of 37° C was maintained using heated air flow.

For each animal, multi-slice localizer images were collected in all three imaging planes (axial, sagittal, and coronal) using a gradient echo sequence with repetition time (TR) = 75ms, echo time (TE) = 5ms, slice thickness = 2mm, flip angle = 35 °, and an average of 4 acquisitions. Additional parameters include field of view (FOV) = 50mm x 50mm and data matrix = 128 x 128.

Following localizer scans, a single-shot, single-slice EPI sequence was used to acquire a CEST spectrum through the approximate midline of the tumor. The saturation period consisted of a series of Gaussian shaped RF pulses at 2.8μT, with a 540° flip angle and 10μs spacing, for a total duration of 5 seconds. A total of 55 saturation frequencies were used, ranging from +10ppm to -4ppm in 0.3ppm increments, and from -4ppm to -10ppm in 1ppm increments, with an additional two scans at -100ppm and +100ppm. Additional parameters include repetition time (TR) = 7.5 seconds, echo time (TE) = 31.8ms, number of experiments (NEX) = 1, slice thickness = 2mm, field-of-view (FOV) = 32mm x 32mm, and matrix = 96 x 96.

**Analysis.** A two-pool model (water at 0ppm and solute at 4.4ppm) Lorentzian fit was used to process the CEST Z-spectra to quantify the CEST effect from the solute. The number of fitted pools and the solute frequency offset were estimated by observing exchange effects on Z-spectra. The fitting was performed to achieve the lowest root mean square (RMS) of residuals between the measured data and model.

## Animal justification.

The goal of the paper was to describe the development of NSPS and VU0945652. The in vivo and in vitro studies were used as proof of concept. Given the large differences in radiotracer, ^18^F-NaF and ^18^F-FDG, uptake in tumors between baseline and post treatment where ^18^F-FDG uptake approached zero post NSPS versus background p<<0.05. While increasing the number of animals would improve the power of the conclusions, it’s unlikely to add to the biological significance of the results. Thus, the use of more animals per tumor model may not be well justified in the context of this publication. All animal studies were approved by our Institutional Animal Care and Use Committee (IACUC), protocol number M1800140, at Vanderbilt University Medical Center (VUMC).

## Alizarin red S and von Kossa staining protocols.

Following euthanasia of the mice used in this study, the tumors were harvested and fixed in 10% formalin for 24-48 hours. Then the tumors were stained for alizarin red S and von Kossa by the Translational Pathology Shared Resources (TPSR) at VUMC using the attached protocols. The slides were then imaged at up to 40X magnification using a brightfield whole slide imager (Leica SCN400 Slide Scanner) at the Digital Histology Shared Resources at VUMC or at our lab using an Olympus SZ61 microscope (I. Miller Precision Optical Instruments, Inc., Feasterville PA) equipped with a Sony 4K HDMI 8.0 megapixel digital camera. The resulting images of the stained slides were processed using Digital Slide Archive (DSA) software that included an appropriate scale bar in each image at various magnifications or using Live image InFocus software (I. Miller).

The figure below shows sample control slides of von Kossa, alizarin red S, cleaved caspase 3, and Ki 67 received from TPSR and imaged via the Leica SCN400 slide scanner. White arrows point to positive stains.


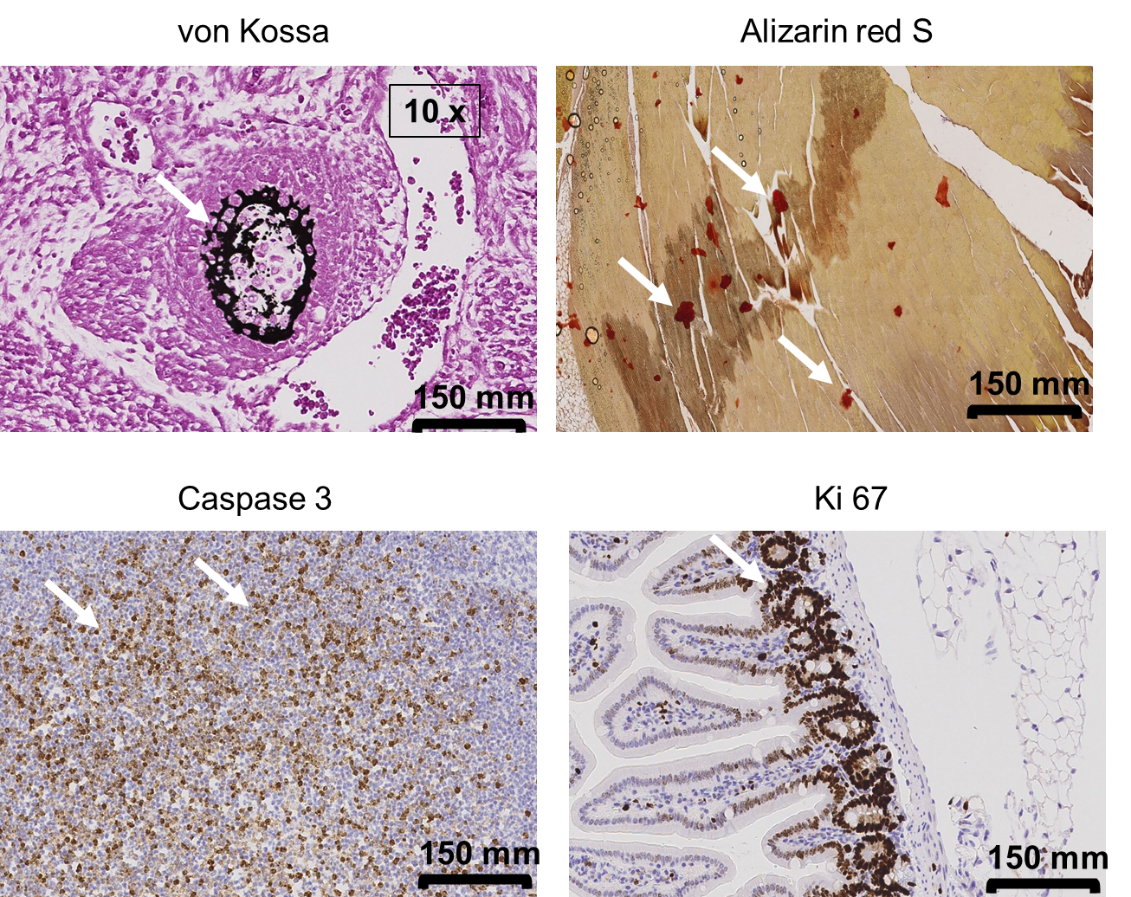


# List of supplementary tables and figures


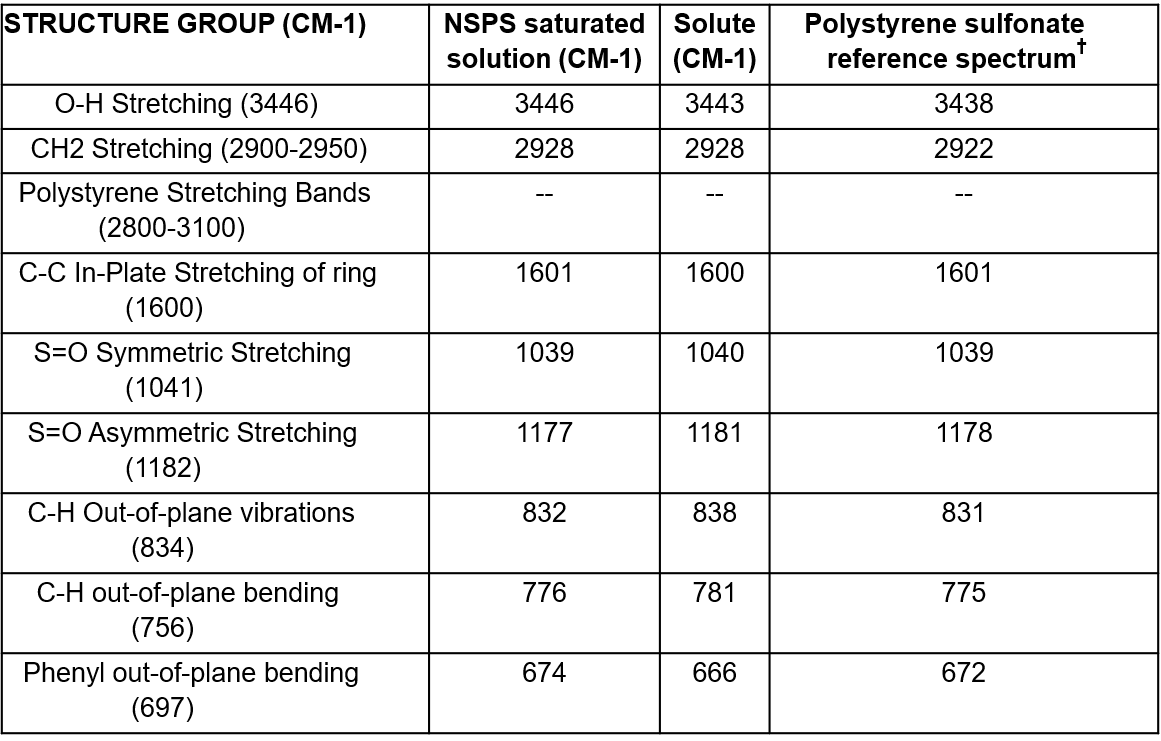
**Table S 1: FT-IR Results for NSPS saturated solution and solute**. The peaks are shown in supplementary figure S4 and are consistent with those of polystyrene sulfonate.

^†^ Brijmohan *et al*.^58^

| **Table S2. Broad applicability of NSPS on mouse models of cancer.** ^18^F-FDG uptake (%ID/g) in tumors of orthotopic mouse models at baseline and within 24 hours post onetime treatment with 100 mg/kg of NSPS. Values displayed as means (SEM). | | |
| --- | --- | --- |
| Tumor model | Baseline | Post NSPS |
| MDA-MB-231 (breast) (n = 6^†^) | 2.63 (0.34) | 0.73 (0.16)  p<0.05 |
| PC3 (prostate) (n = 4) | 3.73 (0.97) | 1.31 (0.20)  p<0.05 |
| HCA7 (colon) (n = 4) | 2.97 (0.66) | 0.86 (0.12)  p<0.05 |
| 4T1 (breast) (n = 5) ^††^ | 1.73 (0.40) | 0.95 (0.18)  p<0.05 |
| H292 (lung) (n = 4) ^†††^ | 2.94 (0.11) | 3.05 (0.41)  p>0.05 |

^†^ In each study, a control cohort consisting of the same number of animals was imaged at baseline and after vehicle (saline) injection. In each study, ^18^F-FDG uptake in the tumor was comparable to baseline values of the experimental cohort and uptake was unchanged following saline injection.

^††^ Baseline ^18^F-FDG in these tumors is lower compared to other tumor models due to the presence of central regions with low FDG uptake as shown in Fig. S7.

^††^ This mouse model was used as negative control due to absence of detectable TME-HAP in the tumors.


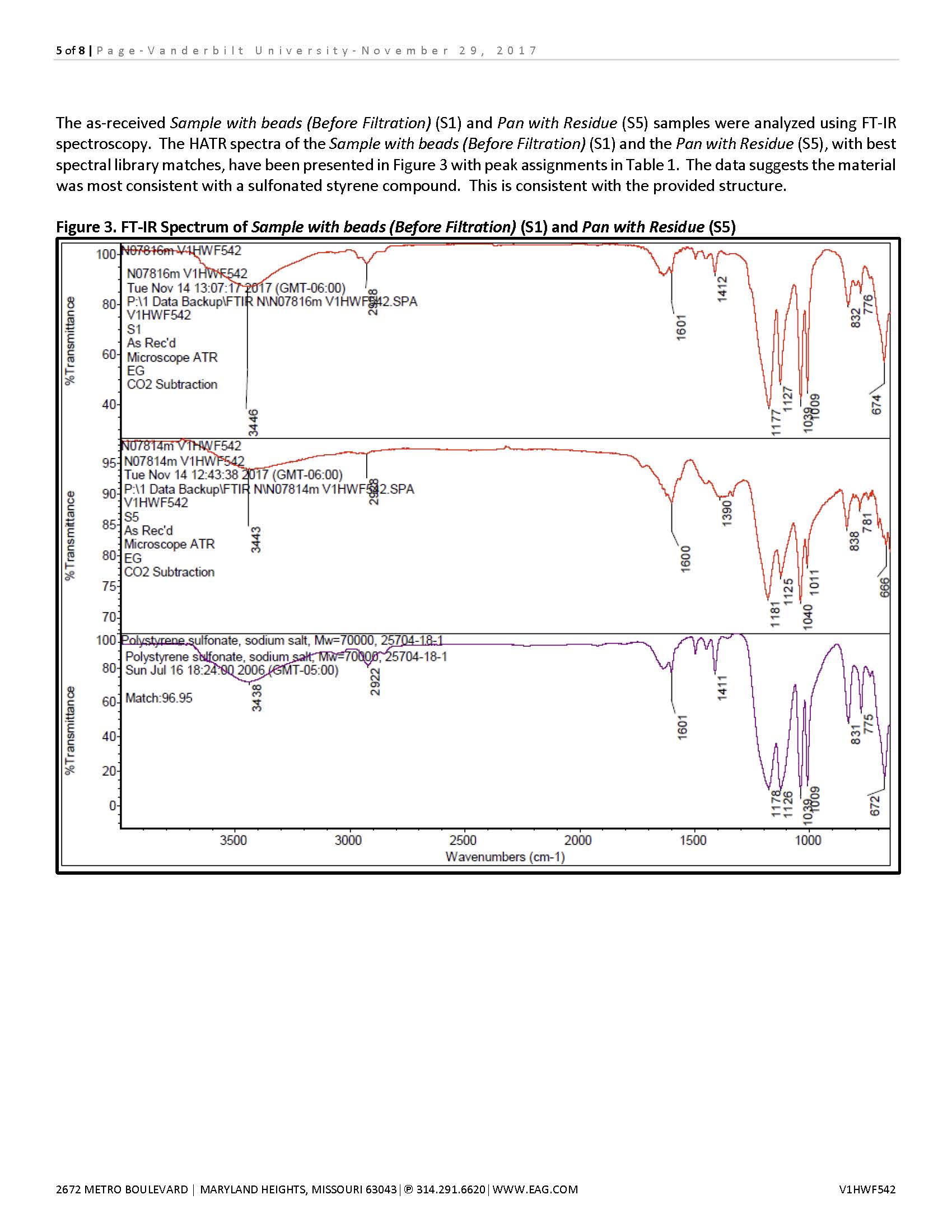
**Figure S 1: NSPS is a polymer similar in characteristic to cation exchange resins.** Spectrum of NSPS solution (top) and solute from NSPS (middle) analyzed by FT-IR spectroscopy at EAG laboratories. The spectra of each sample was compared to a spectral library and is most consistent with a sulfonated styrene compound (bottom spectrum) as expected for NSPS. Peak assignments are in supplementary data Table S1.


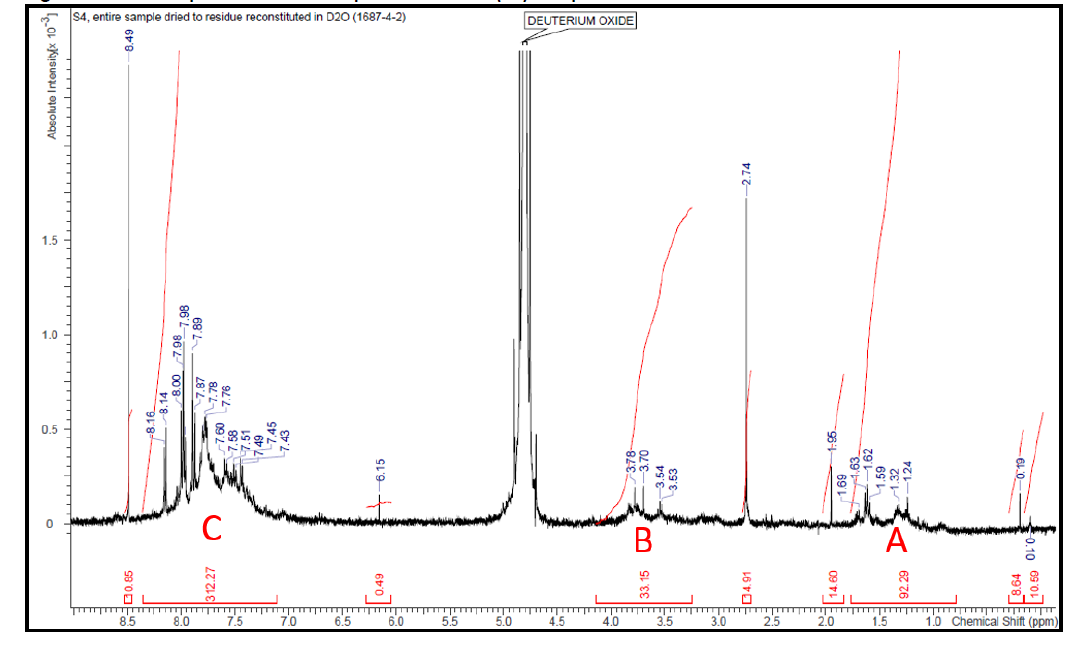


**Figure S 2: 1H NMR Spectrum of NSPS prepared in D2O.** NMR spectrum, analyzed by EAG laboratories (www.EAG.com), includes features consistent with the aliphatic protons on the polymer backbone (A), protons attached to both the aromatic and aliphatic backbone region (B), and the aromatic protons (C).


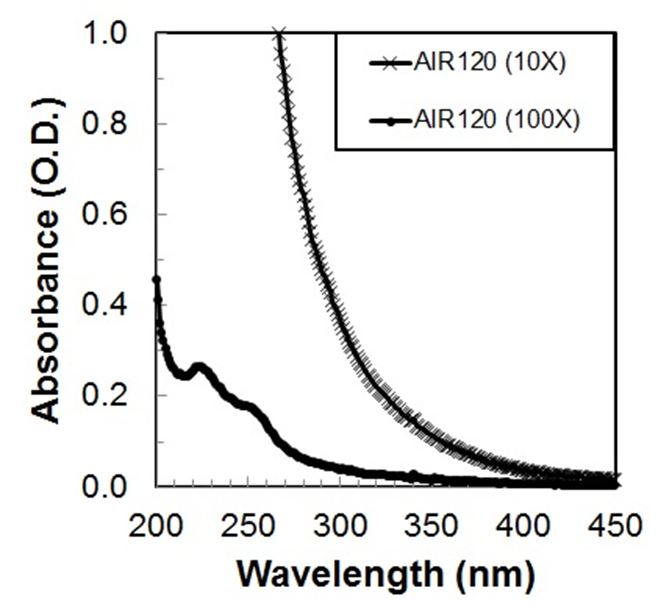


NSPS (10x)

NSPS (100x)

**Figure S 3: Ultraviolet spectrum of NSPS consistent with polystyrene.** Absorbance spectra of NSPS after dilution 10X or 100X into phosphate-buffered saline (PBS). At 10X dilution, the spectrum is dominated by scattering from the colloidal solution; at 100X, absorption features are evident at ~228 nm and ~255 nm consistent with the UV absorption spectrum of polystyrene.


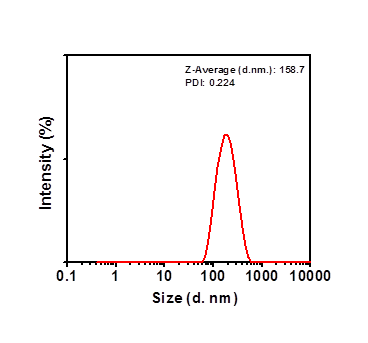
**Figure S 4: Dynamic light scatter (DLS) analysis of NSPS.** Sample preparation which exhibited a z-average nanoparticulate diameter of 159 nm and polydispersity index (PDI) of 0.224.


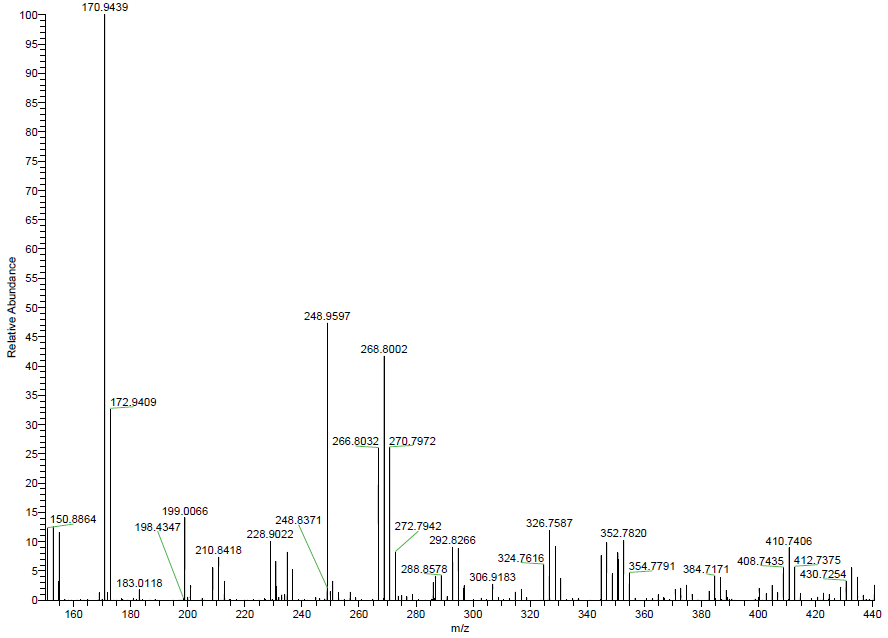

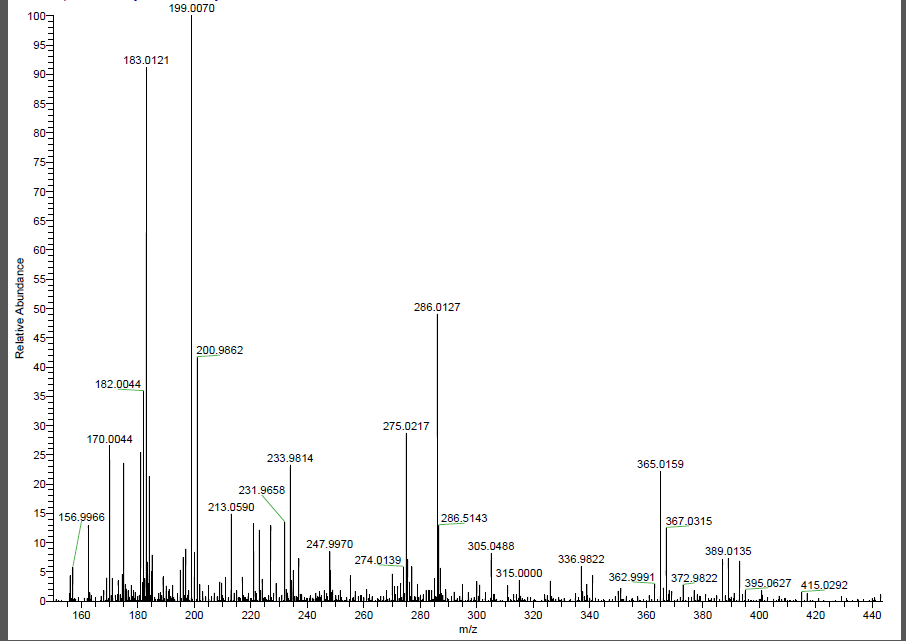


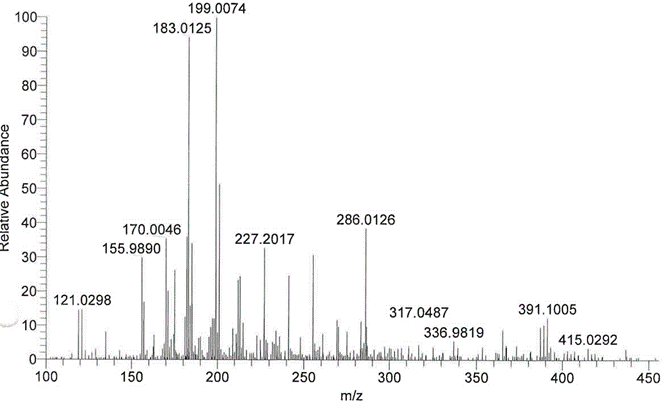


**Figure S 5. NSPS shelf life is at least 1 year.**  Mass spectrum of NSPS stored in a refrigerator for one year (**top**) and two years (**middle**). While the 199 and 183 peaks were observed at 1 year, they were not present in the 2-year spectrum indicating that NSPS loses stability between 1 and years in storage. For references, we show the original spectrum of NSPS (**bottom**) which was already displayed in figure 1.


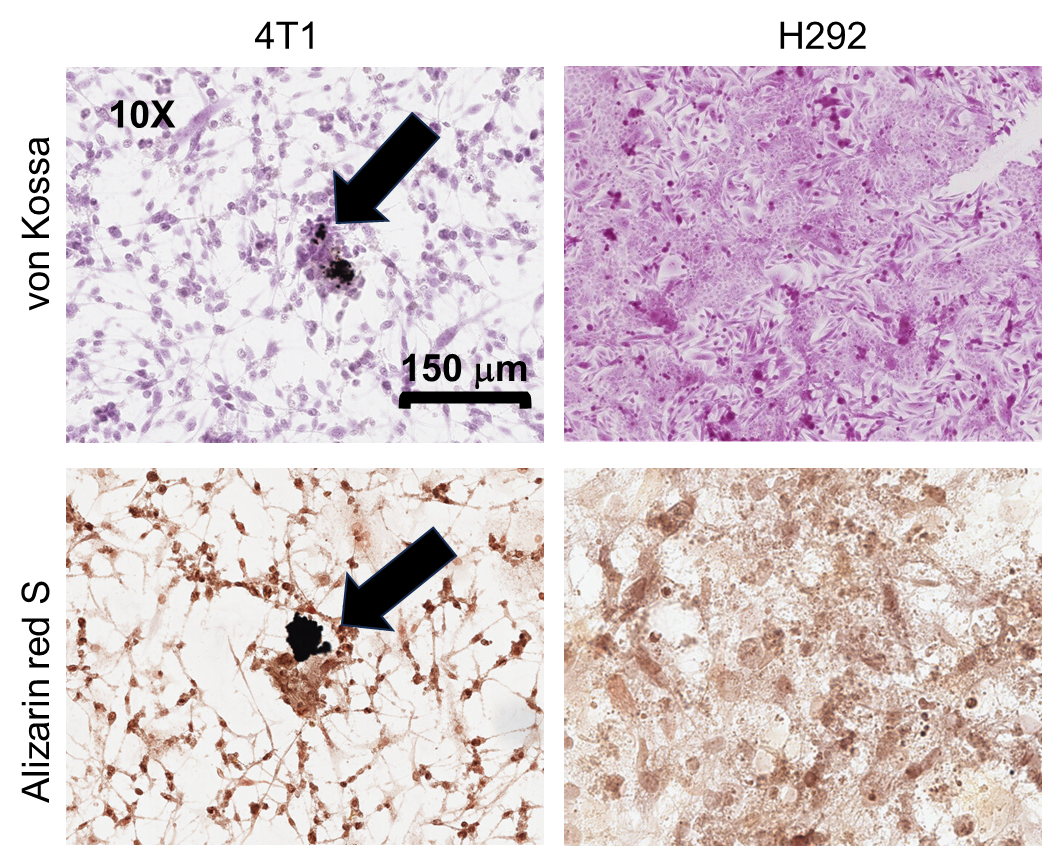


**Figure S 6. Extracellular matrix HAP (ECM-HAP) detectable in 4T1 breast cancer cells but not H292 lung cancer cells, *in vitro*.** Staining with von Kossa and alizarin red S of 4T1 breast cancer cells grown on slides in DMEM median containing osteogenic cocktail for at least 11 days and H292 lung cancer cells in normal DMEM. Arrows point to example of positive stains. Sample MDA-MB-231 stained cells can be found in our previous work in reference 11.


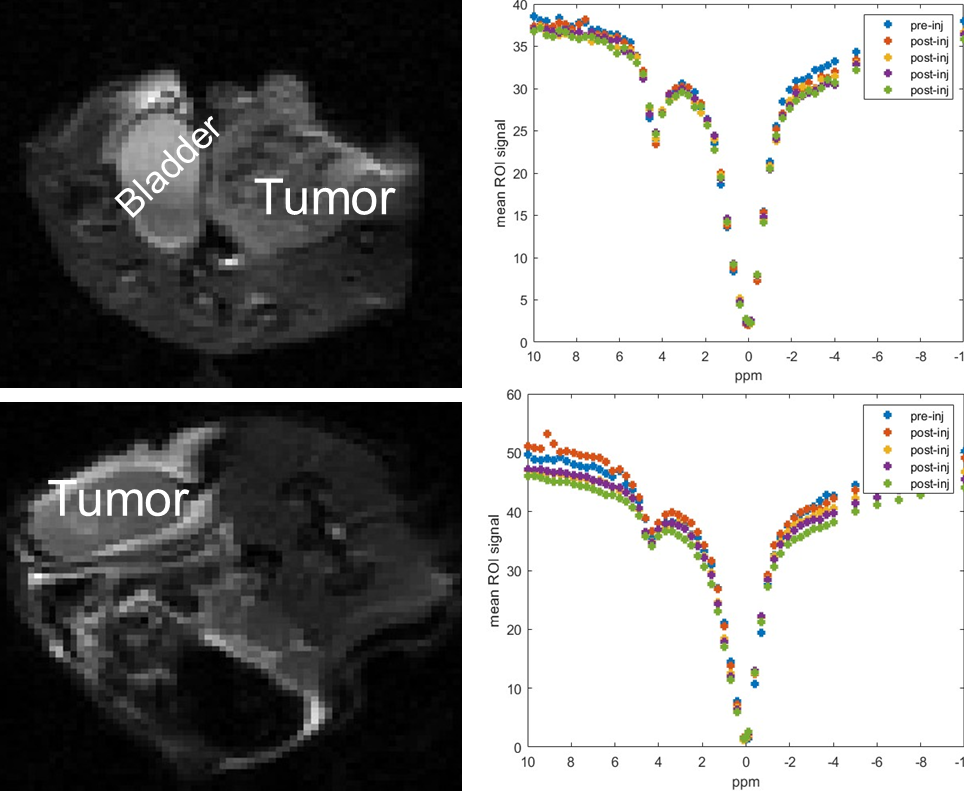


**Figure S 7: little to no changes in pHe of controls or tumors lacking TME-HAP following treatment.** T2 weighed MRI (**left**) and corresponding tumor CEST data (**right**) of a 4T1 breast tumor scanned at baseline and after vehicle (saline) injection (**top panel**) and H292 lung tumor following treatment with 25 mg/kg NSPS (**bottom panel**).

**
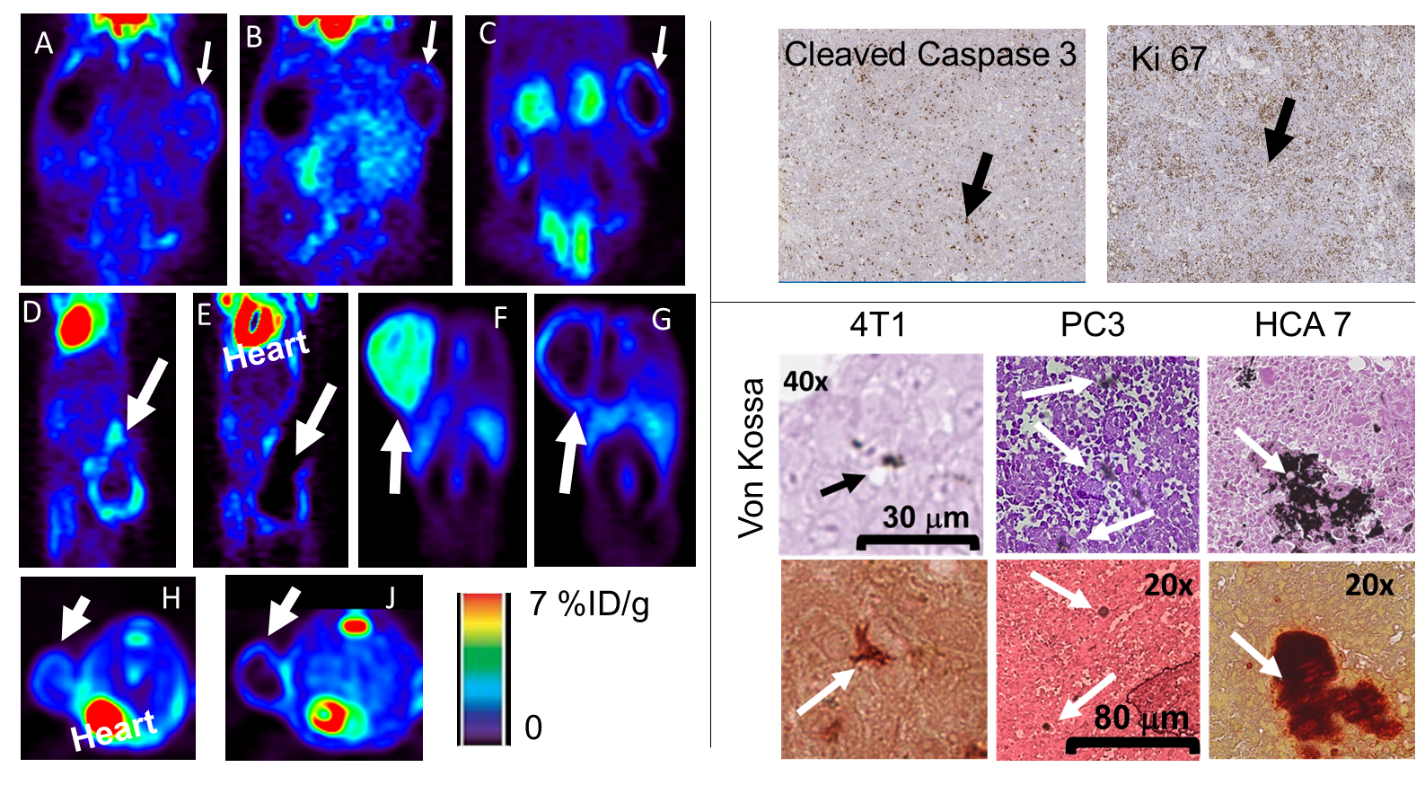
**

**Figure S 8: NSPS inhibits glucose metabolism in many types of tumors with HAP, *in vivo*.** ^18^F-FDG PET images of: (**A**) nude mice with MDA-MB-231 breast tumors imaged at baseline (coronal view) and at (**B**) 24 hours and (**C**) 1 week post treatment NSPS; (**D**) 4T1 tumors in the mammary fat pad at baseline (sagittal view) and (**E**) 24 hrs post NSPS treatment; (**F**) PC3 prostate in nude mice at baseline (coronal view) and (**G**) 24 hrs post NSPS treatment; and (**H**) HCA-7 colon tumors in nude mice at baseline (axial view) and (**J**) 24 hrs post NSPS treatment. NSPS treatment for all mice was one-time 100 mg/kg/0.2 ml injected i.v. A ~75% reduction in FDG uptake was observed in all tumors following NSPS treatment. (**Right panel; Top**) IHC staining samples of cleaved caspase 3 and Ki 67 from harvested HCA 7 tumor section. Arrow points to example of positive stains. (**Right panel; Bottom**) Sample von Kossa and alizarin red S tumor sections. Arrows point to examples of positive stains. Proof of the presence of TME-HAP in MDA-MB-231 tumors was provided in previous work (see references 11 & 12).


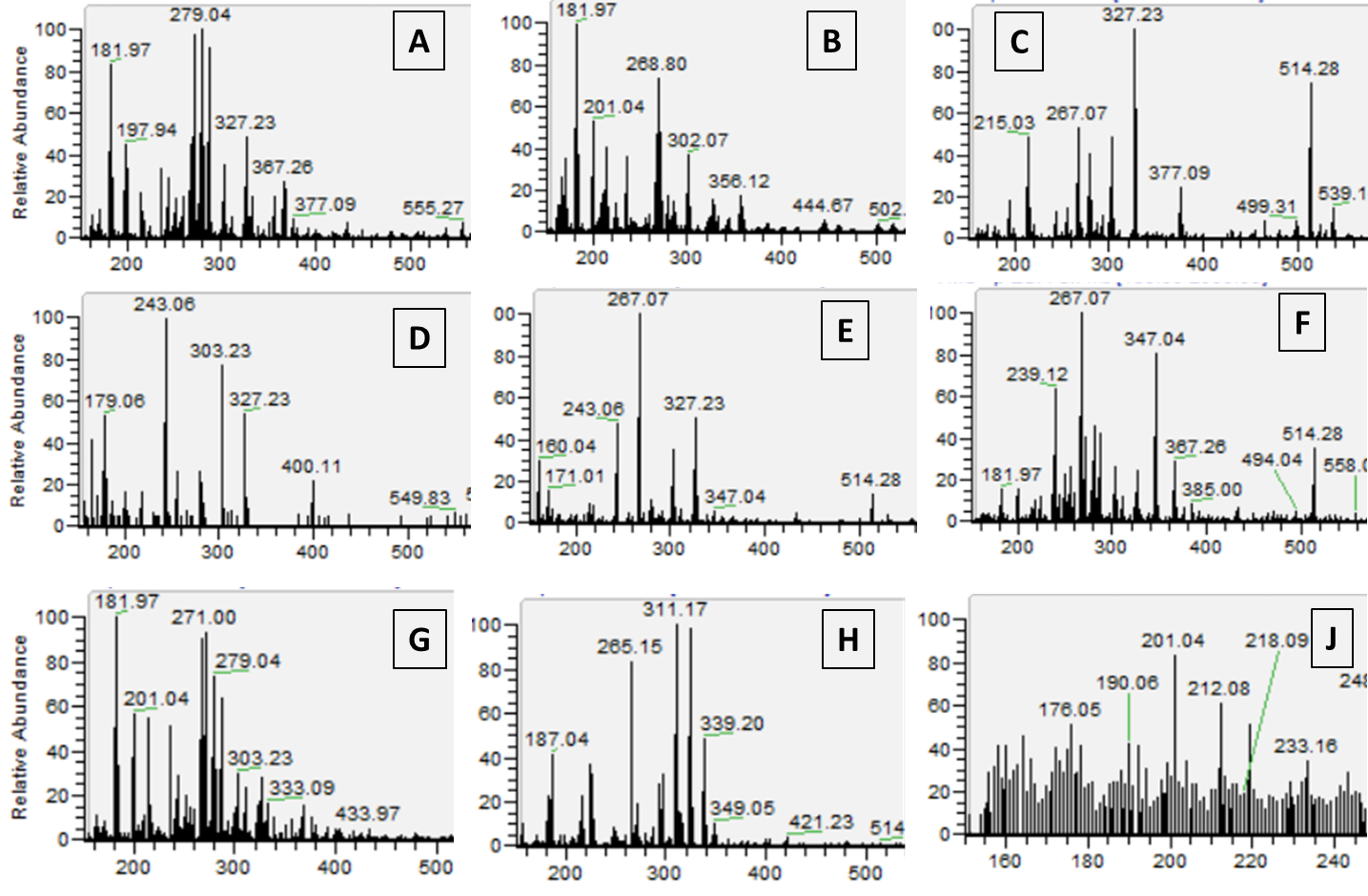
**Figure S 9. NSPS is present in Tumor but absent in normal soft tissue.** Mass spectroscopy of tumor and other organs harvested from white Balb/c mice with 4T1 tumors in the mammary fat pad and injected i.v. with either 100 mg/kg of NSPS where **A)** tumor at 10 min, **B)** tumor at 1 hr. **C)** Liver at 1 hr. **D)** Spleen at 1 hr. **E)** Kidneys at 1 hr. **F)** Bone at 1 hr. **G)** Tumor at 4 hrs. and **H)** bone at 4 hrs or injected with vehicle (saline) where **J)** is harvested 4T1 tumor at 1 hr. NSPS was absent in soft tissue at all the time points tests but appeared to be present in Bone at least during the first hour while absent by 4 hours post NSPS injection.
