# Supplementary material for: Tumor therapy by targeting extracellular hydroxyapatite using novel drugs: A paradigm shift
Source: Cancer Med. 2024 Jan 18;13(3):e6812. doi: 10.1002/cam4.6812 (PMC11025459; doi:10.1002/cam4.6812)
Supplement: Supplementary file 2 — Data S2. [file CAM4-13-e6812-s001.docx]

# Alizarin Red S

Downloaded from WebPath: Internet Pathology Laboratory

**Purpose:** To identify calcium in tissue sections.

**Principle:** Calcium forms an alizarin red S-calcium complex in a chelation process. This reaction is birefringent.

**Control:** A known calcium containing tissue section.

**Fixative:** 10% formalin, or alcoholic formalin.

**Technique:** Cut paraffin sections at 4um.

**Equipment:** Rinse all glassware in DI water: coplin jars, pH meter, bibulous paper, microscope, polarizing lenses.

# Reagents:

Alizarin Red S solution

Alizarin red S…..2.0gm Distilled water…..100.0mL

Mix the solution, adjust the pH to 4.1-4.3 using 0.5% ammonium hydroxide. The pH is critical, make fresh.

Acetone-Xylene

Acetone…..25.0mL Xylene…..25.0mL

# Procedure

1. Deparaffinize and hydrate to 70% alcohol.
2. Rinse rapidly in distilled water.
3. Alizarin red S solution, 30 seconds to 5 minutes, checking microscopically for an orange-red color.
4. Shake off excess dye, blot sections.
5. Acetone-xylene, 20 dips.
6. Clear in xylene, mount in permount.

# Results

Calcium deposits: orange-red

# Notes:

Mouse embryo is good control tissue

*Do not use on de-calicified tissue.*
