# Supplementary material for: Tumor therapy by targeting extracellular hydroxyapatite using novel drugs: A paradigm shift
Source: Cancer Med. 2024 Jan 18;13(3):e6812. doi: 10.1002/cam4.6812 (PMC11025459; doi:10.1002/cam4.6812)
Supplement: Supplementary file 3 — Data S3. [file CAM4-13-e6812-s003.docx]

**Von Kossa’s Method-Calcium Purpose:** To identify the presence of calcium in tissue.

**Principle:** The reaction involved is a chemical one, although it is an indirect way of detecting calcium. The silver reacts with the anions, primarily carbonate and phosphate, of the calcium salts. Bright light reduces the silver salt to metallic silver, and unreduced silver is removed by sodium thiosulfate.

**Fixative:** Alcohols or 10% neutral buffered formalin. Alcohols are preferred.

**Equipment:** Sunlight (preferred) or UV light, chemically clean Coplin jars, graduated cylinders, Erlenmeyer flask

**Control:** Tissue containing known positive calcium deposits, or undecalcified bone.

**Technique:** Cut paraffin sections at 4um.

# Reagents

5% Silver Nitrate

Silver nitrate…..5.0g

Distilled water…..100.0mL

5% Sodium Thiosulfate

Sodium thiosulfate…..5.0g Distilled water…..100.0mL

Nuclear-Fast Red Solution

# Procedure:

1. Deparaffinize and hydrate to distilled water.
2. 5% silver solution, place in bright sunlight, or in front of a 60-watt lamp, place foil (or mirror) behind the jar to reflect the light. Leave for 1 hour or until calcium turns black.
3. Rinse in distilled water, 3 changes.
4. 5% hypo, 5 minutes.
5. Wash in tap water, rinse in distilled.
6. Nuclear-fast red, 5 minutes.
7. Wash in water.
8. Dehydrate, clear, and coverslip.

# Results:

Calcium salts…..black Cytoplasm…..pink Nuclei…..red

# Notes:

1. As noted above, this method detects anions combined with calcium, and not the calcium.
2. Alcoholic iodine solution used form the removal of mercury pigment may also remove some of the calcium salts form the tissue.
3. Most texts state that calcium stains black with von Kossa’s reaction and that has been our experience; however Meloan and Puchtler state that, as emphasized by von Kossa, the black deposits in this reaction are due to a reduction of silver by organic matter in the sections. The black reaction product is an artifact that occurs only when the sections are exposed to strong light. If the reaction is carried out in subdued light, yellow to yellowish-brown silver phosphate is selectively demonstrated. Silver carbonate is soluble in sodium thiosulfate and is not demonstrate.
4. Luna notes that problems encountered with this technique may be due to the use of unbuffered formalin (formalin pigment will reduce silver), the use of artificial light (produces a brown reaction product), or inadequate exposure to the silver solution.
